# Integrated remote sensing and geochemical data of Shadli mineralized metavolcanics (Egypt): mantle plume-driven magmatism during subduction–rift transition

**DOI:** 10.1038/s41598-026-60562-2

**Published:** 2026-07-14

**Authors:** Mohamed Zaki Khedr, Mahmoud A. Sayed, Shehata Ali, Mokhles K. Azer, Akihiro Tamura, Tomoaki Morishita, Eiichi Takazawa, Yuji Ichiyama, Ali M. Mahdi

**Affiliations:** 1 Department of Geology, Faculty of Science, Kafrelsheikh University, Kafrelsheikh, 33516 Egypt; 2 Geology Department, Faculty of Science, Qena University, Qena, 83523 Egypt; 3 Geology Department, Faculty of Science, Minia University, El-Minia, 61519 Egypt; 4 Geological Sciences Department, National Research Centre, Cairo, Egypt; 5 Department of Earth Sciences, Kanazawa University, Ishikawa, 920-1192 Japan; 6 Geology Department, Faculty of Science, Niigata University, Niigata, Japan; 7 Graduate School of Science, Chiba University, 1-33 Yayoi-Cho, Inage-Ku, Chiba, 263-8522 Japan

**Keywords:** Bimodal volcanism, magmatism during subduction–rift transition, Mineral and whole-rock chemistry, Mantle-plume contribution, Remote sensing, Southern Eastern Desert of Egypt, Planetary science, Solid Earth sciences

## Abstract

The Wadi Ranga–Atshan metavolcanic (WRAM) suite in the Southern Eastern Desert of Egypt is part of the Shadli calc-alkaline metavolcanic belt, which is the main crustal part of the Arabian-Nubian Shield (ANS). Integrated remote sensing, mineral compositions, and whole-rock chemical data are used to elucidate the mantle-plume contribution, petrogenesis, and geodynamic evolution of the ~ 739 Ma Shadli metavolcanics. Remote sensing analysis using Landsat-8 and ASTER band ratios in RGB effectively discriminates between the dominant felsic and mafic varieties (bimodal volcanism) and minor intermediate types along with the different alteration zones (e.g., iron- and Al‒OH-rich zones). Its PCA and CEM techniques are used to delineate phyllic, argillic, and propylitic alteration zones and their associated Cu–Fe–Zn sulfides, iron oxides, and malachite mainly along the NW–SE shear zones. The whole rock chemistry indicates that mafic rocks (metabasalts) and felsic to intermediate types (metarhyolites, metadacites, and metabasaltic andesites) show tholeiitic and calc-alkaline natures, respectively. But alkali metabasalts exhibit alkaline affinity. The calc-alkaline metavolcanic protoliths are derived from 10–20% partial melting of a depleted spinel lherzolite mantle source, supported by their low REEs (ΣREEs < 20 ppm) contents. They are further characterized by enrichment in LILEs and depletion in HFSEs (Nb < 2.6, Ta < 0.08, and Ti < 53.31 ppm), suggesting a typical arc-related magmatic signature. In contrast, the alkali metabasalt protoliths are derived from low partial melting (~ 5% melting) of the enriched garnet lherzolite or garnet–spinel lherzolite source in deeper and enriched mantle parts, supported by their high REEs (ΣREEs: 212 ppm) contents. The alkali metabasalts are strongly enriched in HFSEs (Ti > 14,676, Nb > 28.9, and Ta > 1.7 ppm) along with high Cr (up to 337.8 ppm) and Ni (up to 245.1 ppm) relative to calc-alkaline types, consistent with intraplate (OIB-like type) magmatism due to upwelling of mantle plume. The investigated metavolcanics plot in MORB–arc and within-plate/OIB fields, reflecting coexistence of OIB-like (mantle plume-derived melts) and arc/MORB-like basaltic melts, together with arc-like magma signatures; these results indicate tectonomagmatic evolution of the studied rocks from supra-subduction arc magmatism to intraplate rifting transition during the arc assembly of the ANS. Therefore, the different partial melting, magmatic affinity, and magma source (mixed plume–arc magmas) possibly reflect plume-driven magmatism during the subduction–rift transition of the ANS. This transitional environment may be characterized by a sequence of magmatic sulfide mineralization (disseminated pyrite and chalcopyrite) to post-magmatic hydrothermal alteration and supergene oxidation assemblages (gossans, talc–carbonate, malachite, and chrysocolla). These processes were structurally controlled by NW–SE (Najd-related) and NE–SW shear zones that developed during the subduction–rift transition, which facilitated mantle-derived magmatism and fluid migration, thereby generating volcanogenic massive sulfide (VMS) mineralization. The arc-related hydrothermal systems, plume-related ore systems, and later metasomatic overprints may highlight the metallogenic significance of the ANS.

**Supplementary Information:**

The online version contains supplementary material available at 10.1038/s41598-026-60562-2.

## Introduction

The Arabian-Nubian Shield (ANS) constitutes the majority of NE Africa (Nubian Shield) and the Arabian Peninsula (Arabian Shield), covering about 3 × 10^6^ km^2^ of the total area. It was formed by the breakup of the Rodinia supercontinent (870–800 Ma)^1,2^ due to the opening of the ancient Mozambique Ocean^3^. This was followed by a convergence movement that led to the subduction of the oceanic lithosphere of the Mozambique Ocean, resulting in island arc and back-arc terranes^2,4^. Recently, several studies, especially for ore exploration, have been done because the ANS is enriched in precious metals (e.g., Au, Ag, Cu, Pb, and Zn). Intensive exploration and mining activities are ongoing in the ANS, targeting gold, copper-lead–zinc mineralization, and other metals associated with volcanogenic massive sulfides (VMS).

**Fig. 1:**
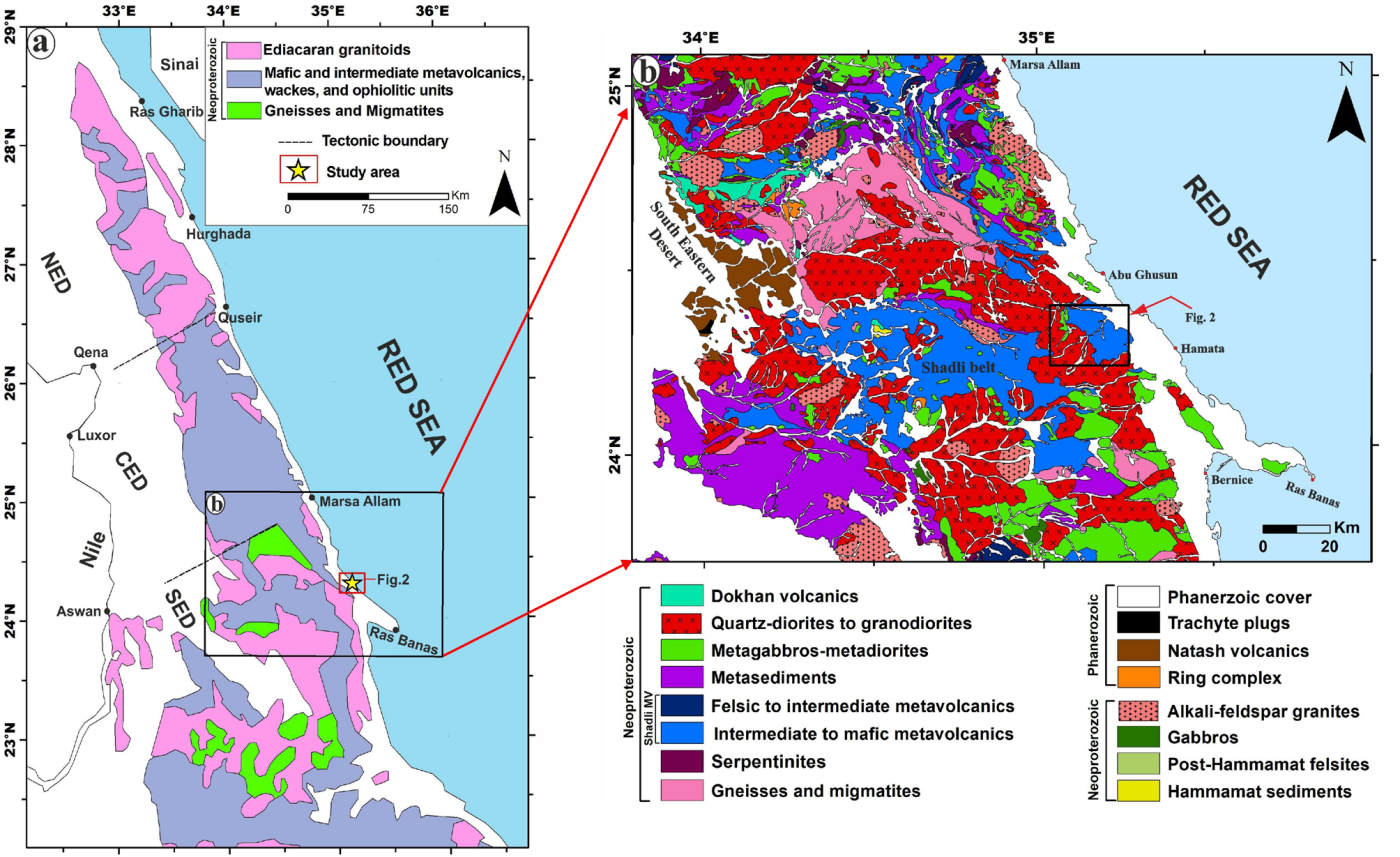
Simplified geologic maps show (**a**) the distribution of Neoproterozoic basement rocks and the location of the study area in the Eastern Desert of Egypt (modified after Stern and Hedge^8^), (**b**) the distribution of the Shadli metavolcanics in the Southern Eastern Desert of Egypt (modified after Conoco^9,10^, EGSMA^11,12^; and related aerial photographs). The inset of Fig. 2 is shown. Abbreviations: NED; North Eastern Desert, CED; Central Eastern Desert, SED; Southern Eastern Desert. This figure was created by ArcGIS Desktop 10.8 https://www.esri.com/en-us/arcgis/products/arcgis-online).

The Neoproterozoic metavolcanics in the Eastern Desert (ED) are classified into two suites: older metavolcanics (OMV; ~ 830–685 Ma) and younger metavolcanics (YMV; ~ 800–670 Ma)^5,6^. The OMV rocks are related to ophiolites and comprise mainly pillowed tholeiitic basalts, while YMV (or arc metavolcanics) rocks have a calc-alkaline affinity with high K. The YMV are generated in an island arc setting, which is dominant in the Southern Eastern Desert (SED), including the studied Wadi Ranga–Atshan metavolcanics (WRAM) (Fig. 1a). The island arc associations in the SED are composed of a sequence of arc metavolcanics (e.g., Shadli metavolcanics) and their derived metasediments, along with their corresponding intrusive rocks such as tonalites, diorites, and intrusive metagabbros^7^.

The Shadli-Ranga belt (SRB) (Fig. 1b) has been intruded by Hamata post-collisional granitoids, dividing it into the eastern part of the Ranga suite and the Shadli suite. The latter suite is considered the most substantial entity of all Neoproterozoic metavolcanics in the Nubian Shield^13^. The tectonic setting of the Shadli metavolcanics is a subject of ongoing debate, with three principal views proposed. The first emphasizes an arc-related origin, supported by arc-like geochemical signatures^14,15^, and further linked to formation in a back-arc basin environment^16,17^. The second advocates a continental rift setting or an intra-arc extensional regime^18^. The third highlights a plume-driven scenario, in which the metavolcanics are attributed either to plume-related rift magmatism^19^ or to the involvement of a plume-modified lithospheric mantle^20^.

The investigated WRAM in the SED (Fig. 1a) are part of the Shadli-Ranga belt (SRB) (Fig. 1b), which is distinguished by the simultaneous occurrence of both felsic and mafic varieties^15,21^. This SRB is interpreted as a Late Tonian–Cryogenian mantle-plume–related hotspot track developed in the juvenile Arabian–Nubian Shield^20^. New U–Pb zircon geochronology, zircon O–Hf isotopes, and whole‑rock Sr–Nd isotopes for metavolcanic rocks in the SRB yield concordia ages of 739 ± 3.4 Ma and show tholeiitic, calc‑alkaline, and high‑K calc‑alkaline affinities, indicating mixed plume–arc magmas^20^. The SRB is composed of both mafic and felsic volcanic rocks, including metabasalt, metaandesite, metadacite, metarhyolite, and associated volcaniclastic rocks^14,18^. There is still a conventional controversy among petrologists related to the genetic association of those end members^22–24^. The mafic metavolcanics indicate magma originating from the mantle, while the felsic ones originated from the partial melting of the lower crust by ascending mantle-derived mafic melt^14,25,26^. In addition, felsic metavolcanics in the SRB may be formed from the same magma source as the mafic endmembers as a result of immiscibility or crystal fractionation of their mantle-derived parent source^27–29^.

The metavolcanics in the SRB are rich in volcanogenic massive sulfide (VMS) deposits, i.e., the Um Samiuki-Abu Hamamid prospect^30,31^. The VMS (Fe–Cu–Zn–S) in the studied Shadli metavolcanics is the main type of sulfide mineralization in the SED of Egypt^17,32^. Three main hypotheses for the origin of this sulfide mineralization include (i) a reaction between the melt and the volcanic wall rocks in a hydrated environment^33,34^, (ii) the instant deposition and stabilization of hydrothermal fluids after crystallization of volcanic rocks^35^, and (iii) the subsequent redistribution and remobilization of circulating hydrothermal fluids during the extensive deformation along shear zones^32,36,37^. This third one possibly explains the origin of the Fe–Cu–Zn–S in the WRAM.

The studied WRAM were described as Shadli metavolcanics with low K‐calc-alkaline affinity^38^, similar to low-K tholeiitic bimodal characters of basalt-rhyolite associations of Gabal Sarubi and Wadi Dendikan^15,39^. The present study deals with the integration of remote sensing and geochemical data, including mineral chemistry (major, trace, and REE), EDS–SEM, and whole-rock chemistry (major, trace, and REE) of the WRAM-hosted Fe–Cu–Zn–S mineralization. The main aim is to shed light on mantle plume-driven magmatism and associated mineralization during subduction–rift transition and to understand petrogenesis and geodynamic evolution of Shadli bimodal metavolcanics as a main crustal part of the ANS. This study also discusses the factors controlling the arc-related Fe–Cu–Zn–S mineralization in the crustal part of the ANS and the effect of metasomatism, including element mobility and enrichments during post-magmatic stages.

## Geologic setting

**Fig. 2:**
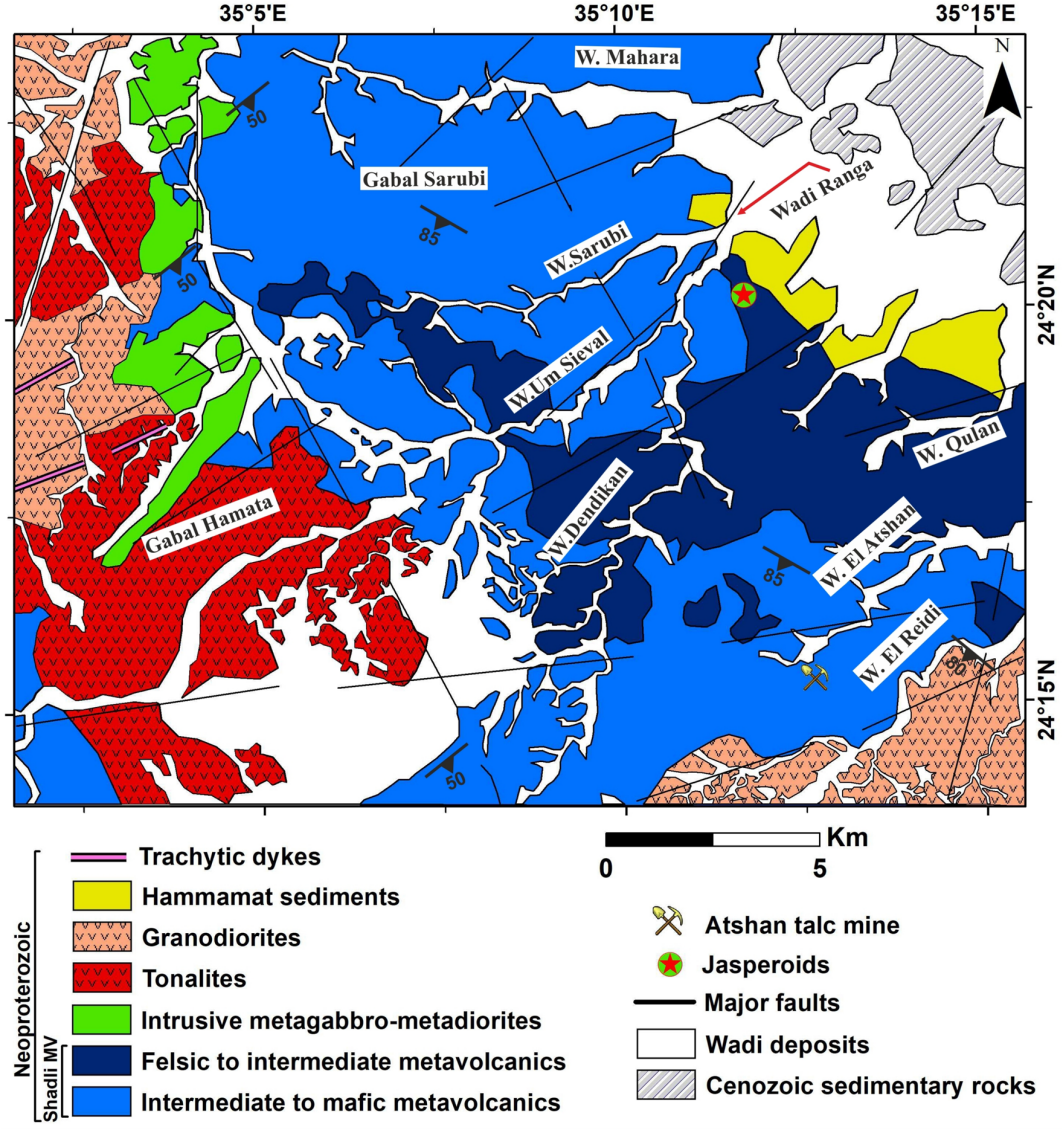
A detailed geologic map of the WRAM area based on remote sensing data and field verification shows the different rock units, structures, jasperoids, and Wadis in the study area. This figure was created by ArcGIS Desktop 10.8 (https://www.esri.com/en-us/arcgis/products/arcgis-online).

The WRAM is located ~ 90 km south of Marsa Allam City and ~ 20 km north of the Hamata area along the Halayeb-Shalateen road and bordered by latitudes 24° 23′ 25.7″ to 24° 23′ 33″ N and longitudes 35° 15′ 39″ to 35° 15′ 40.6″ E (Figs. 1b and 2). The area covers about 250 km^2^ of the Neoproterozoic basement rocks and is bounded to the east by both Hammamat sediments and the Phanerozoic sedimentary succession at the coastal plain of the Red Sea (Fig. 2). The Wadi Ranga area extends to the north until it meets Gabal Sarubi metavolcanics, which were previously investigated for malachite and different Cu mineralizations^40^. Wadi Ranga, cutting through the investigated metavolcanic rocks, includes three tributaries named Wadi Sarubi, Wadi Um Seival, and Wadi Dendikan, respectively, from the north to the south, and all of them show trending from the east to the west^9,11^. The Wadi Ranga metavolcanics are extended to the north through Gabal Sarubi and to the south through Wadi Dendikan (Fig. 2). Metavolcanics of Gabal Sarubi and Wadi Dendikan are comparable to Shadli metavolcanics in the SED and exhibit bimodal characters of basalt-rhyolite associations^15,39^. In addition, the Wadi Atshan runs through the southeast part of the studied metavolcanics. It includes one of the famous talc mines, named the Atshan talc mine (Fig. 2).

The studied WRAM exhibits distinct features of multiple phases of extensional and compressional structural events related to the Neoproterozoic Pan-African Orogeny (900–550 Ma)^3,41^. Field observations and analysis of aerial photographs indicate that the Wadi Ranga area is predominantly controlled by NW–SE and NE–SW structural trends (Fig. 2), manifested by shear zones, thrust faults, and major dykes that crosscut the metavolcanics. The predominant NW–SE fault sets intersect with NE–SW structural trends, accompanied by a minor E–W set. Talc and malachite mineralization are restricted along these shear zones. The NW–SE trend corresponds to the Najd fault system, a regionally extensive fault system that affects the Arabian–Nubian Shield and has significantly influenced its tectonic evolution, structural deformation, and the localization of mineral deposits (Fig. 2).

**Fig. 3:**
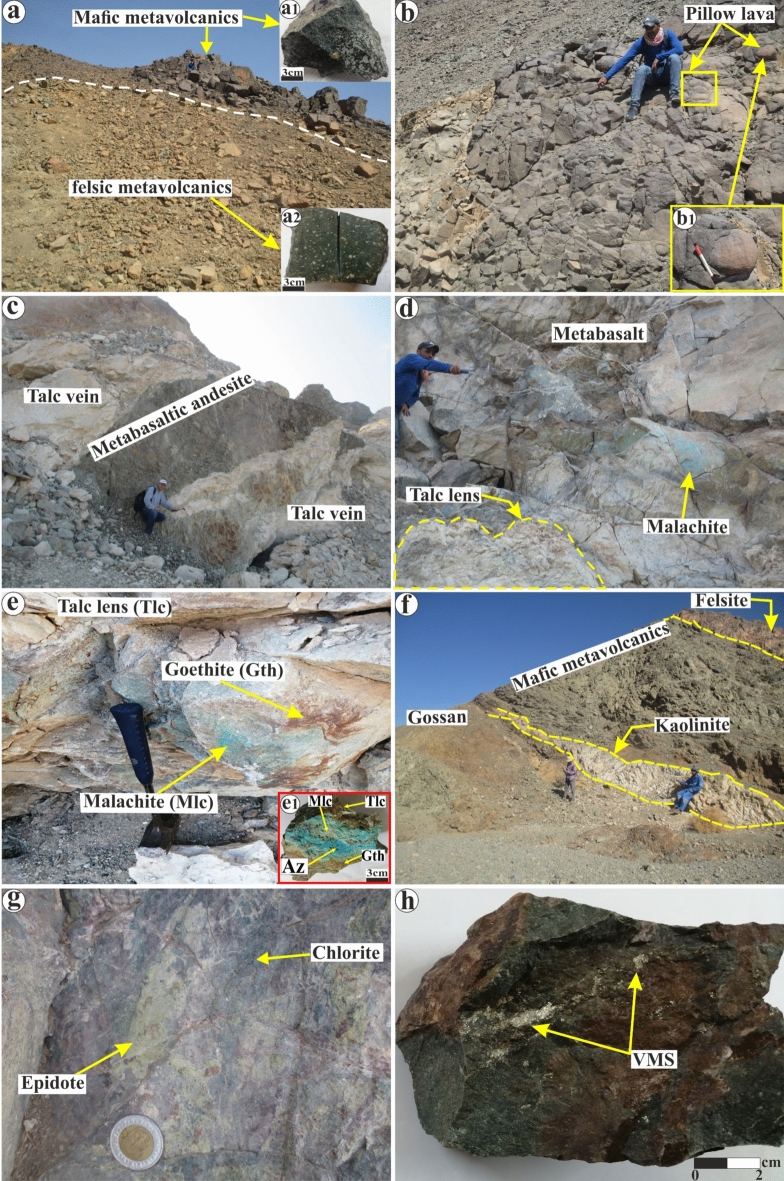
Field photographs and hand specimens show important geologic features of the WRAM area. (**a**) contact between the mafic and felsic metavolcanics, (**b**) pillow basaltic lavas at the Wadi Sarubi with a zoom-in inset in “b1”, (**c**) talc vein hosted in metabasaltic andesite at the Atshan talc mine, (**d**) talc lens and malachite hosted in metabasalt at the Atshan talc mine, (**e**) talc-bearing malachite associated with goethite alteration, the inset of “e1” refers to a malachite-azurite-bearing talc sample from the mine, (**f**) gossan, kaolinite alteration zones, and felsite dyke in mafic metavolcanics, (**g**) epidote and chlorite alteration appearing over some metavolcanics outcrops, (**h**) volcanogenic massive sulfides within a mafic rock sample. Abbreviations: metavolcanics (MV), malachite (Mlc), talc (Tlc), azurite (Az), goethite (Gth), and volcanogenic massive sulfides (VMS).

The Neoproterozoic rocks around the Wadi Ranga area comprise metavolcanics, syn-orogenic granites, a metagabbro-metadiorite complex, late- to post-tectonic granites, and Hammamat sediments^15,42,43^. Wadi Ranga metavolcanic rocks are intruded by the metagabbro-diorite complex in the northwest and by Hamata granitoids in the south and southwestern parts (Fig. 2). Previous studies suggest that the WRAM are of bimodal composition, which comprises moderate- to high-relief mafic and felsic varieties comparable with those of the Shadli belt^15,20,39^. The fieldwork of the Ranga metavolcanics indicates that they comprise mainly intermediate to mafic varieties, where felsic to intermediate types are less abundant (Fig. 2). The former covers the greater central part of the study area, whereas the latter is found in the southeast region near the Atshan talc mine (Figs. 2 and 3a).

The mafic varieties are low-grade metamorphosed, dark greenish-gray basalts, alkali basalts, and basaltic andesites associated with lapilli tuffs and agglomerates (Fig. 3a). Moreover, they reveal amygdaloidal textures with voids filled with chlorite and carbonates, which are commonly epidotized. Pillow structures are recorded at the entrance of the Wadi Sarubi, ranging in size from 50 cm up to 1 m across (Fig. 3b, b1). These typical pillow lavas of metaandesite or basaltic metaandesite compositions suggest submarine eruption, possibly in an island-arc setting. The felsic varieties, which outcropped near Wadi Qulan El-Atshan, Wadi Dendikan, and some at Wadi Ranga, are characterized by light yellow to buff colors and comprise weakly metamorphosed porphyritic rhyolite, dacites, and related pyroclastics (Fig. 3a). The felsic rocks are bordered by Hammamat sediments to the east and by the mafic metavolcanics in the northern and southern parts (Fig. 2).

The Atshan area comprises both felsic and mafic varieties (metarhyolites, metadacite, metabasaltic andesites, metabasalts, and a few metaandesites) with abundant pockets of talc, tremolite talc, and talc carbonate rocks distributed along E–W, NW–SE, and NE-SW shear zones that crosscut the WRAM. The Atshan talc mine contains two varieties of talc: massive talc veins and small talc lenses. The former is widely distributed and is hosted by the metaandesites to metabasaltic andesites (Fig. 3c), while the latter is observed in limited parts of the area and is hosted by the metabasalts (Fig. 3d). The Atshan mineralized metavolcanics host talc and malachite-azurite-bearing talc bodies, which occur commonly close to or along the shear zones (Fig. 3d and e).

Furthermore, the study area shows evidence of mineralization along alteration zones (e.g., gossan and kaolinization) within the mafic metavolcanics associated with felsite dykes (Fig. 3f). The gossan occurs as an irregular, rusty-brown, iron-rich surficial cap (4 m across) formed by supergene oxidation of sulfide-bearing rocks. In contrast, kaolinite appears as a continuous, whitish, vein-like body (7 m length-2 m width), which crosscuts the metavolcanics and extends concordantly along shear zones. Additionally, the study area reveals significant features of metasomatism, such as epidote and chlorite alteration in some metavolcanic outcrops in the Wadi Ranga area (Fig. 3g). Lenticular bodies of red jasperoids up to 2 m in thickness and more than 10 m in length are seen at the entrance of Wadi Ranga^43^. These lenticular bodies are rich in sulfide mineralization, which is mainly represented by pyrite. Moreover, the metavolcanics host a stockwork of quartz veinlets, mainly associated with volcanogenic massive sulfides (VMS), which are observed in the field, not so far from the gossans and alteration zones (Fig. 3h).

## Methodology

For a detailed geological mapping of the Wadi Ranga area, Landsat-8 Operational Land Imager (OLI) and the Advanced Spaceborne Thermal Emission and Reflection Radiometer (ASTER) satellite images (Supplementary Table 1a, b) are used to discriminate rock units, structures, and alteration zones associated with copper and sulfide mineralization (for remote sensing techniques, see Supplementary Tex. 1). The automatic lineament extraction tool was employed to determine the common structural trend that controls the mineralized zones.

Thirty rock samples from Wadi Ranga metavolcanics were selected for major element analysis (Supplementary Table 2), using X-ray Fluorescence (XRF) at the Geo Analytical Lab, Washington University, USA (for analysis conditions, see Supplementary Tex. 1). Additionally, some trace elements and REE (Supplementary Table 2) were obtained using Inductively Coupled Plasma Mass Spectrometry (ICP–MS) at the Geo Analytical Lab, Washington University, USA (Supplementary Tex. 1).

The major element analyses (Supplementary Table 3a) of silicate minerals were performed by using EPMA (JEOL JXA-8230) at Chiba University (for analysis conditions, see Supplementary Tex. 1). Additionally, the in-situ determination of trace and REE concentrations (Supplementary Table 3c) in plagioclase, amphibole and clinopyroxene from the WRAM was carried out using Laser-ablation inductively coupled plasma mass spectrometry (LA–ICP–MS) at Kanazawa University, Japan (for analysis conditions, see Supplementary Tex. 1). In addition, comprehensive qualitative and semi-quantitative analyses of sixty points, encompassing Fe–Cu–Zn sulfide minerals, Fe–Ti oxides, copper-bearing talc and associated minerals (Supplementary Table 3b), were performed utilizing Scanning Electron Microscopy (SEM) equipped with Energy Dispersive Spectrometry (EDS) at Niigata University, Japan (for analysis conditions, see Supplementary Tex. 1).

## Results

### Remote sensing data

**Fig. 4:**
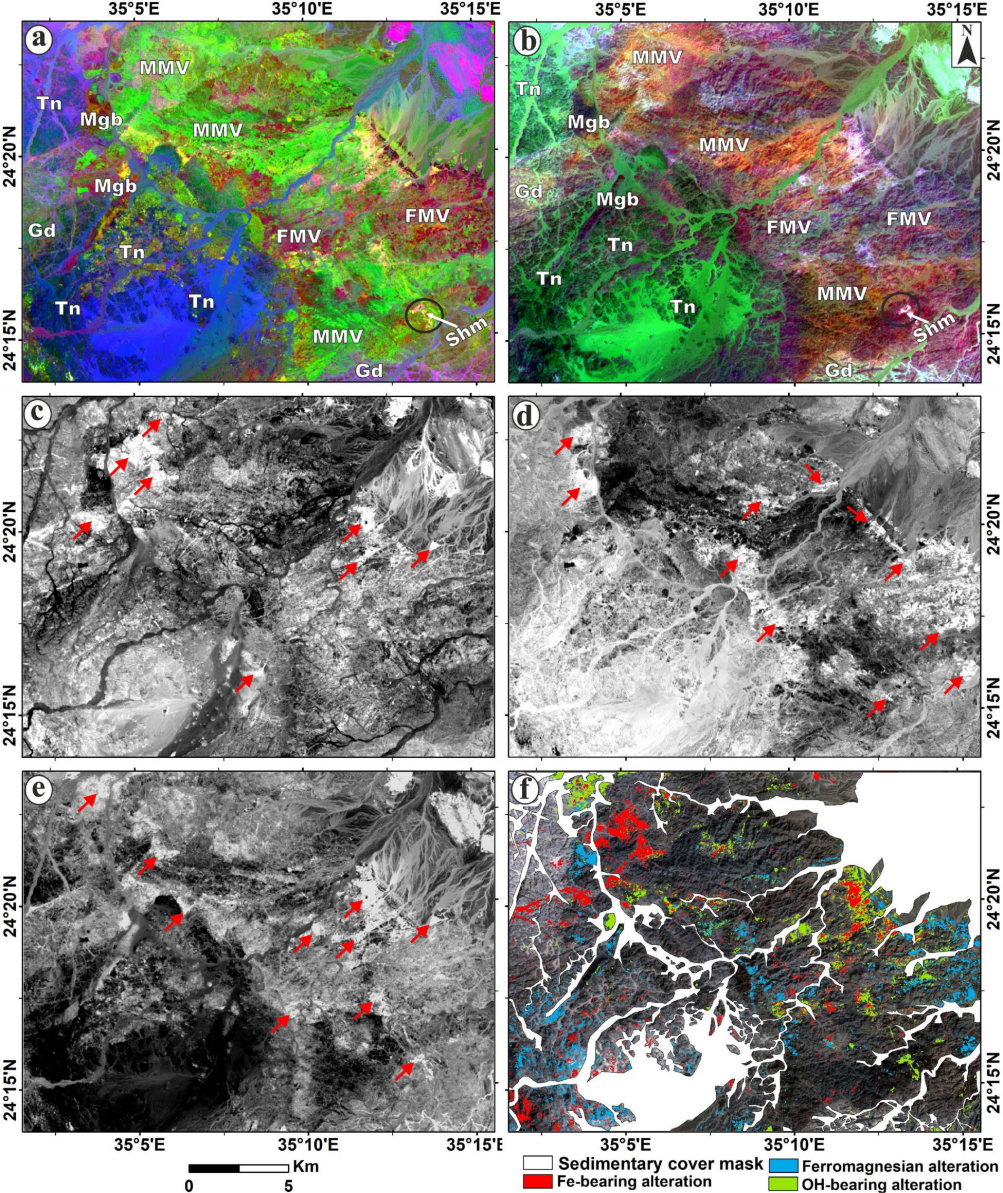
(**a**) Composite Landsat-8 BR (b6 /b7, b6/b5*b4/b5, b5) in RGB, (**b**) composite ASTER BR Image (b4 /b8, b4 *b3 /b8, b4/b6) in RGB; (**c**) greyscale Landsat-8 BR (b4 /b2) Image, (**d**) greyscale Landsat-8 BR (b5 /b6) Image, (**e**) greyscale Landsat-8 BR (b6 /b7) Image, (**f**) greyscale density slicing landsat-8 image after building a sedimentary cover mask. Abbreviations: felsic to intermediate metavolcanics (FMV), intermediate to mafic metavolcanics (MMV), metagabbro-diorite (Mgb), granodiorite (Gd), tonalite (Tn), Atshan talc mine (Shm). This figure is generated by Envi 5.3 software (https://www.nv5geospatialsoftware.com/) and exported by ArcGIS Desktop 10.8 software (https://www.esri.com/en-us/arcgis/products/arcgis-online).

**Fig. 5:**
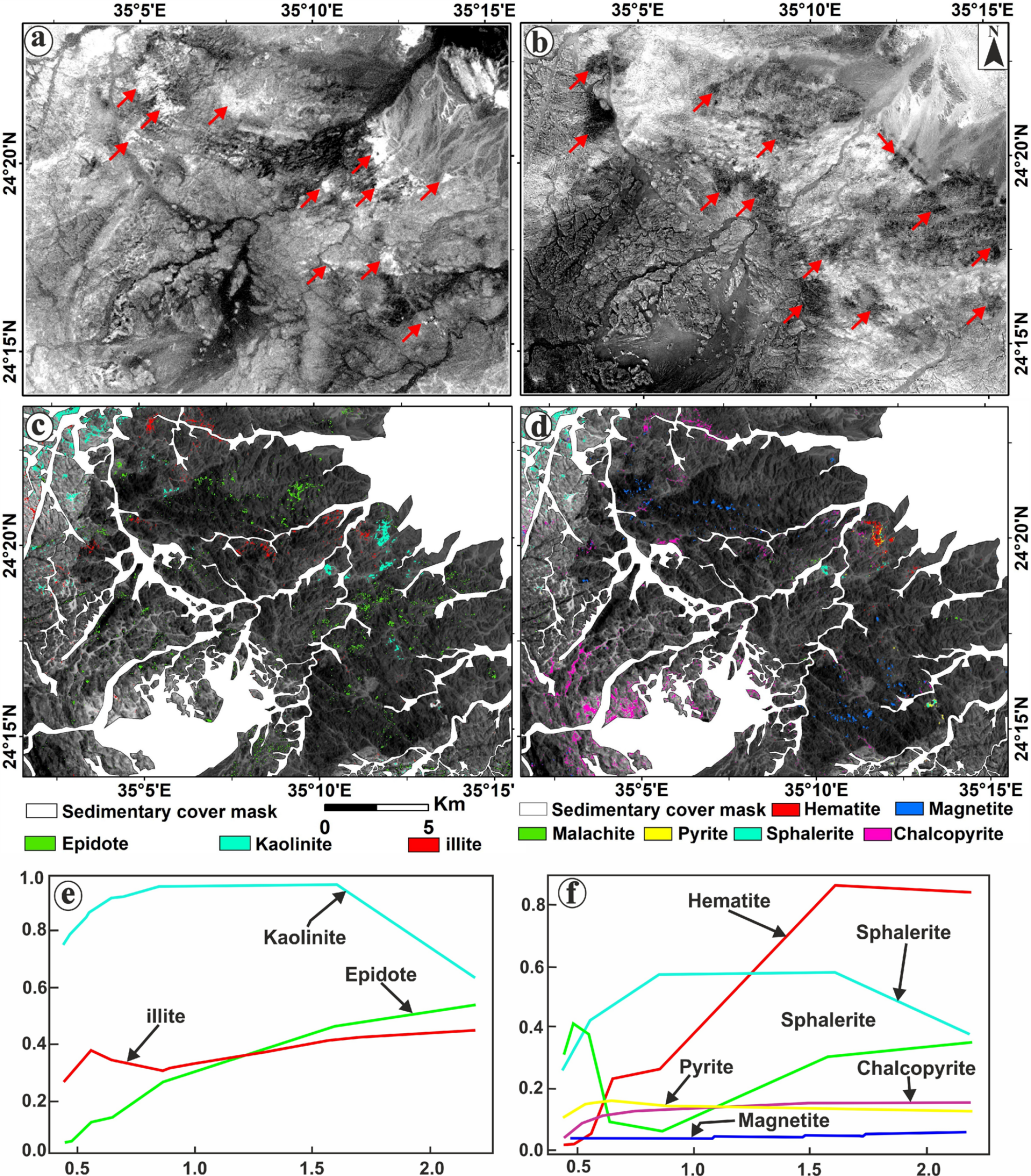
(**a**) PC4 greyscale ASTER Image, (**b**) PC3 greyscale ASTER Image, (**c**) CEM classification endmember for alteration minerals (illite, kaolinite, and epidote) using VNIR-SWIR Landsat-8 data and the USGS resampled spectral library, (**d**) CEM classification endmember of valuable minerals associated with alteration zones using VNIR-SWIR Landsat-8 data and the ASTER resampled spectral library, (**e**), (**f**) Endmember collection spectra used in CEM spectral classification method using USGS spectral library and ASTER spectral library for VNIR-SWIR bands. Solid colored lines symbolize the Landsat-8 bands’ wavelength. This figure is generated by Envi 5.3 software (https://www.nv5geospatialsoftware.com/) and exported by ArcGIS Desktop 10.8 software (https://www.esri.com/en-us/arcgis/products/arcgis-online).

The different remote sensing techniques, such as Band Ratio (BR) and Principal Component (PC), have been used for the discrimination of rock units and alteration zones (Figs. 4 and 5). Additionally, the Constrained Energy Minimization (CEM) and density slicing approaches were used for the best discrimination of rock units and alteration zones in the Ranga-Atshan area (Figs. 4 and 5). Furthermore, the automatic lineament extraction tool was employed to determine the common structural trends that control the mineralized zones (Supplementary Fig. 1a). All former techniques contribute to the optimal enhancement and discrimination of the various rock units in the Ranga-Atshan area, encompassing felsic to mafic metavolcanics, a metagabbro-diorite complex, granodiorite, tonalite, and talc rocks (Fig. 4a, b). These techniques are crucial for delineation of alteration zones, including gossans, phyllic, argillic, and propylitic alterations associated with mineralization, i.e., sulfide, talc, and malachite (Figs. 4 and 5).

BR transformation proves valuable in mapping lithological and hydrothermal alteration zones^44–47^. This method involves dividing the Digital Number (DN) values of a specific band by the corresponding DN values of another band, then presenting the resulting DN values in a grayscale image, thereby conveying relative band intensities^48^. The Landsat-8 BR b6 /b7, b6/b5*b4/5, and b5 in RGB distinguishes Ranga–Atshan felsic to intermediate metavolcanics (FMV) with a dark red color, while the intermediate to mafic ones (MMV) have green. Granitic rocks appear greenish violet for granodiorites (Gd) and bluish violet for tonalites (Tn). The small bodies of metagabbro-diorite (Mgb) around Wadi Ranga–Atshan are stained with reddish brown (Fig. 4a). Similarly, the ASTER b4 /b8, b4*b3 /b8, and b4/b6 BR in RGB distinguish the former rock units with violet, brownish orange, light violet, green, and dark violet, respectively (Fig. 4b). Application of 4/2, 6/5, and 6/7 BR on the Landsat-8 image may distinguish iron oxides, ferromagnesian, and hydroxyl-bearing alteration zones in the Ranga–Atshan area, respectively^44,49,50^. The iron oxides, ferromagnesian, and OH-bearing alteration zones are individually delineated and appear with a white tone in Fig. 4c–e, respectively. The obtained threshold values using statistical results and the density slicing approach are used for best highlighting of the former three alteration zones (4/2, 6/5, 6/7) (Fig. 4f).

Principal component analysis (PCA) and its eigenvector loadings enhance mineral detection, where the sign and magnitude indicate whether a mineral appears as bright or dark pixels in specific PC images^51,52^. Eigenvector loadings of all nine ASTER bands are presented to identify the highest PC image, containing the spectral information related to the examined alteration minerals. High ASTER eigenvectors of bands 2 and 4 in PCs give useful spectral information about iron oxides^53,54^.

The Al(OH)-rich minerals, such as alunite, kaolinite, illite, and muscovite, show distinctive information in bands 5, 6, and 7, while bands 8 and 9 ASTER data give well-informed data about Fe–Mg (OH)-bearing minerals (e.g., chlorite) and carbonate minerals (e.g., calcite and dolomite) ^55,56^. The ASTER eigenvalue of PC1, PC2, and PC3 accounts for 99.13% of the total information (Supplementary Table 1b). The Al(OH)-rich minerals give high reflectance at PC4 (0.574) and distinguish white pixels (Fig. 5a), while iron oxide-bearing alterations show high absorption at PC3 (-0.677); therefore, iron-rich rocks can appear with dark pixels (Fig. 5b).

The CEM approach has become an effective technique for mapping targets^57^. It linearly defines the desired target signatures and minimizes unreliable signatures depending on the selected endmembers. The partial unmixing matrix depends on the assessment of target correlation, which delineates the target anomalies. The variation between the spectra of the target and the endmember background is highlighted by maximizing the signatures of the desired endmembers and restraining the other signatures of the composite unknown background. Moreover, CEM is a helpful approach for mapping the different alteration zones in the office without any need for more field observations^58,59^. The USGS spectral library^60^ was used to designate three alteration minerals (illite, kaolinite, and epidote) from the VNIR/SWIR surface reflectance data (Fig. 5c). The three alteration minerals are good indications of phyllic, argillic, and propylitic alteration zones in the studied Ranga-Atshan, respectively^61,62^. Furthermore, the ASTER spectral library is also used for mapping the possible occurrence of valuable minerals associated with mineralization zones from VNIR-SWIR data. Based on this technique, the investigated valuable minerals such as malachite, chalcopyrite, pyrite, hematite, magnetite, and sphalerite are possibly recognized by using CEM spectrums (Fig. 5d). The wavelengths of the selected endmember collection spectra used in the CEM spectral classification method are shown in Fig. 5e and f. The CEM results are validated by field observations (Fig. 3d–h; malachite, gossans, goethite, and visible sulfides) and also further supported by petrographic (Fig. 7; pyrite, chalcopyrite, and sphalerite) and SEM–EDS data (Supplementary Fig. 2; Supplementary Table 3b), which are discussed in subsequent sections.

**Fig. 6:**
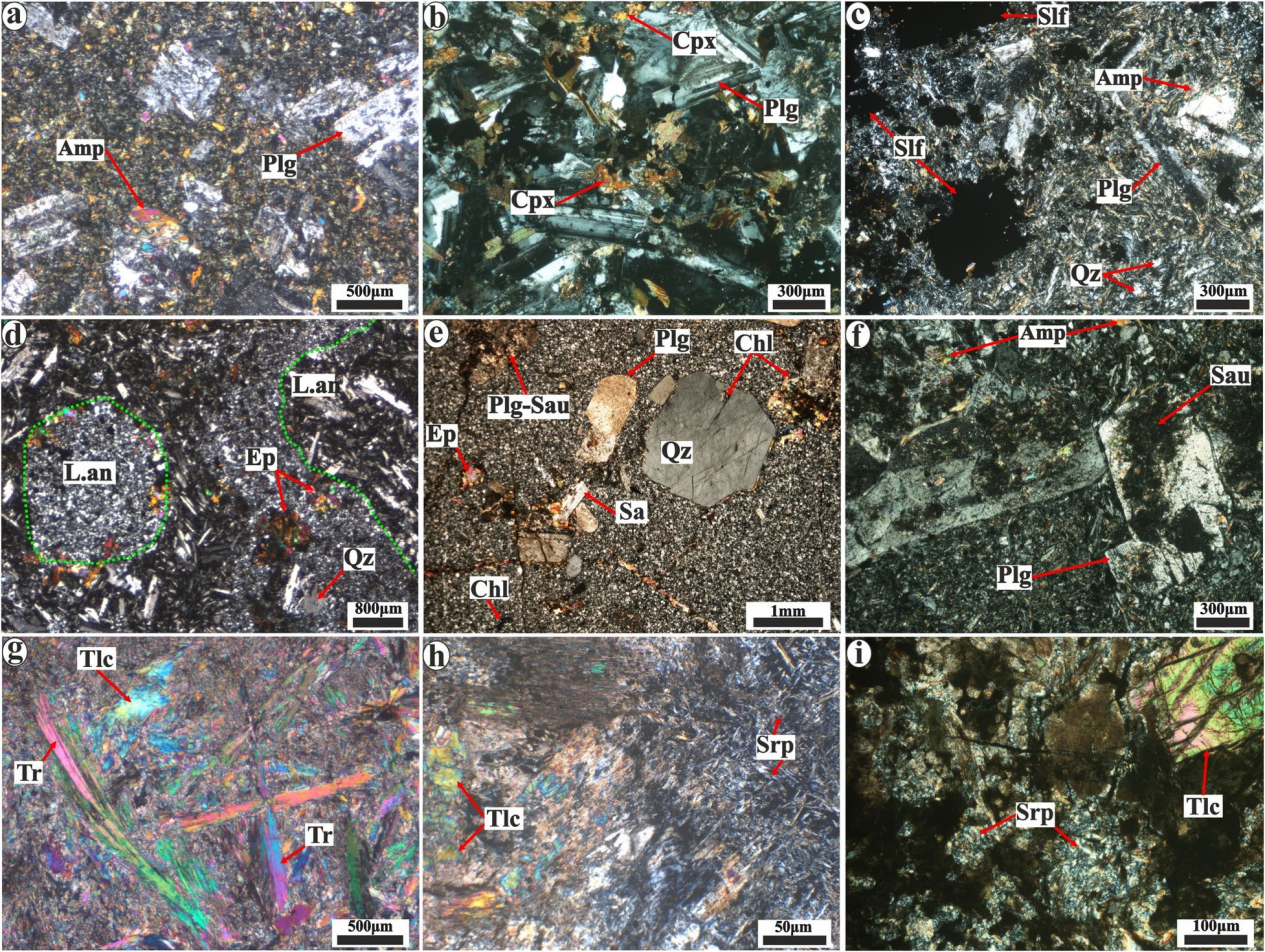
Petrography of the WRAM metavolcanics and related rocks (XPL). (**a**) tabular plagioclase phenocrysts and fine acicular amphibole in metabasalts, (**b**) plagioclase and clinopyroxene in alkali-metabasalts, (**c**) plagioclase microlaths associated with fine quartz in sulfide-bearing metabasaltic andesites, (**d**) Lithic lapilli tuff; (**e**) plagioclase and quartz phenocrysts in metarhyolites, (**f**) saussuritization of plagioclase phenocrysts in Atshan metabasaltic andesites, (**g**) tremolite talc from Atshan mine, (**h**), (**i**) talc rocks associated with serpentine minerals. Abbreviations: plagioclase (Plg), sanidine (Sa), quartz (Qz), amphibole (Amp), clinopyroxene (Cpx), sulfides (Slf), lithic andesite (L.an), epidote (Ep), chlorite (Chl), saussurite (Sau), talc (Tlc), and serpentine (Srp).

**Fig. 7:**
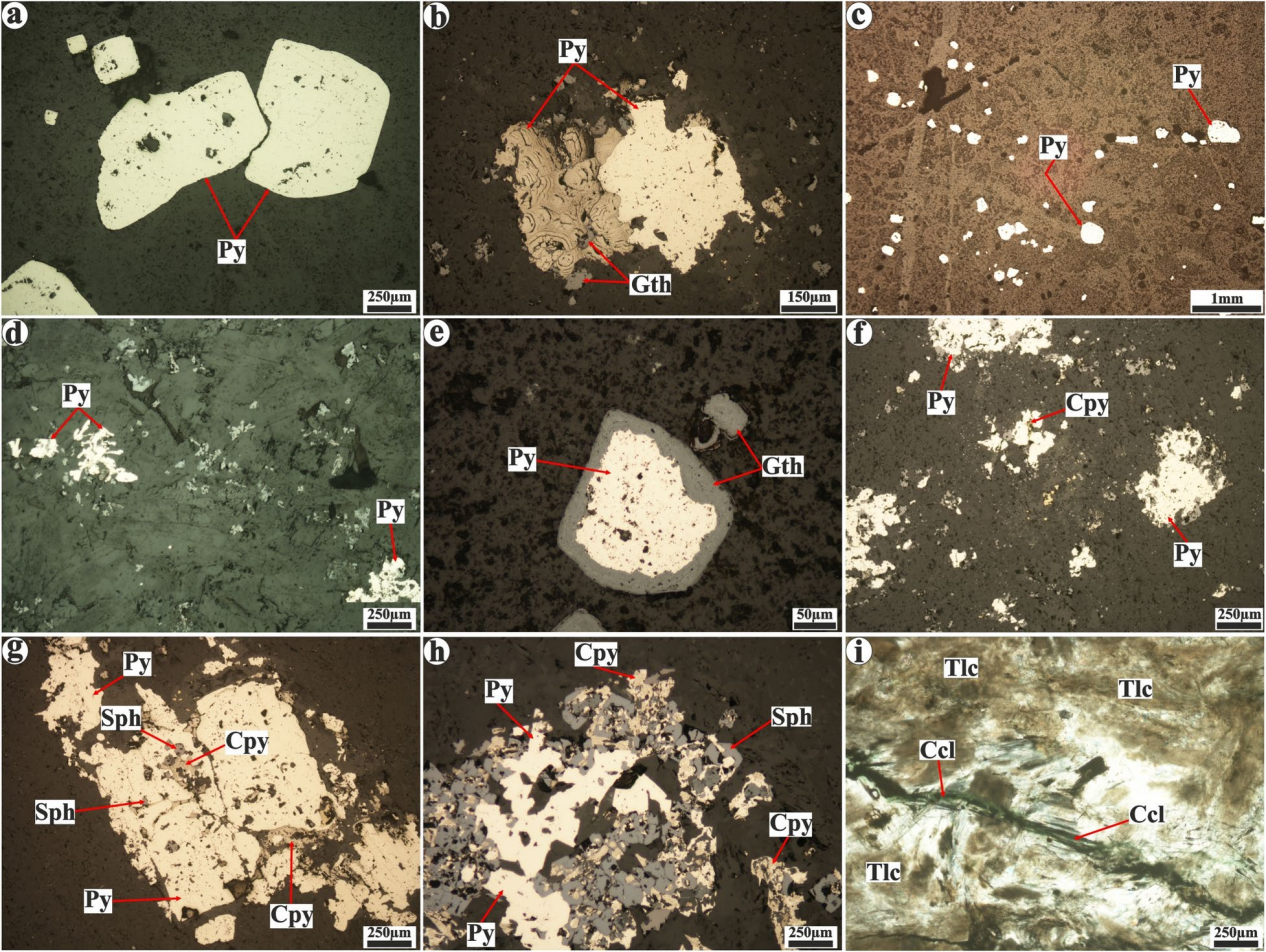
Photomicrographs of the disseminated and massive sulfide mineralization in the WRAM show representative mineral assemblages and textures. (**a**) subhedral cubic pyrite grains, (**b**) anhedral colloform pyrite, (**c**), (**d**) disseminated pyrite, (**e**) concentric rims of goethite envelope pyrite, (**f**) small patches of chalcopyrite replaced pyrite, (**g**), (**h**) large subhedral pyrite crystal partially replaced by patches of chalcopyrite and sphalerite, (**i**) chrysocolla veinlet hosted in the talc rocks. Abbreviations: pyrite (Py), chalcopyrite (Cpy), sphalerite (Sph), goethite (Gth), talc (Tlc), and chrysocolla (Ccl).

**Fig. 8:**
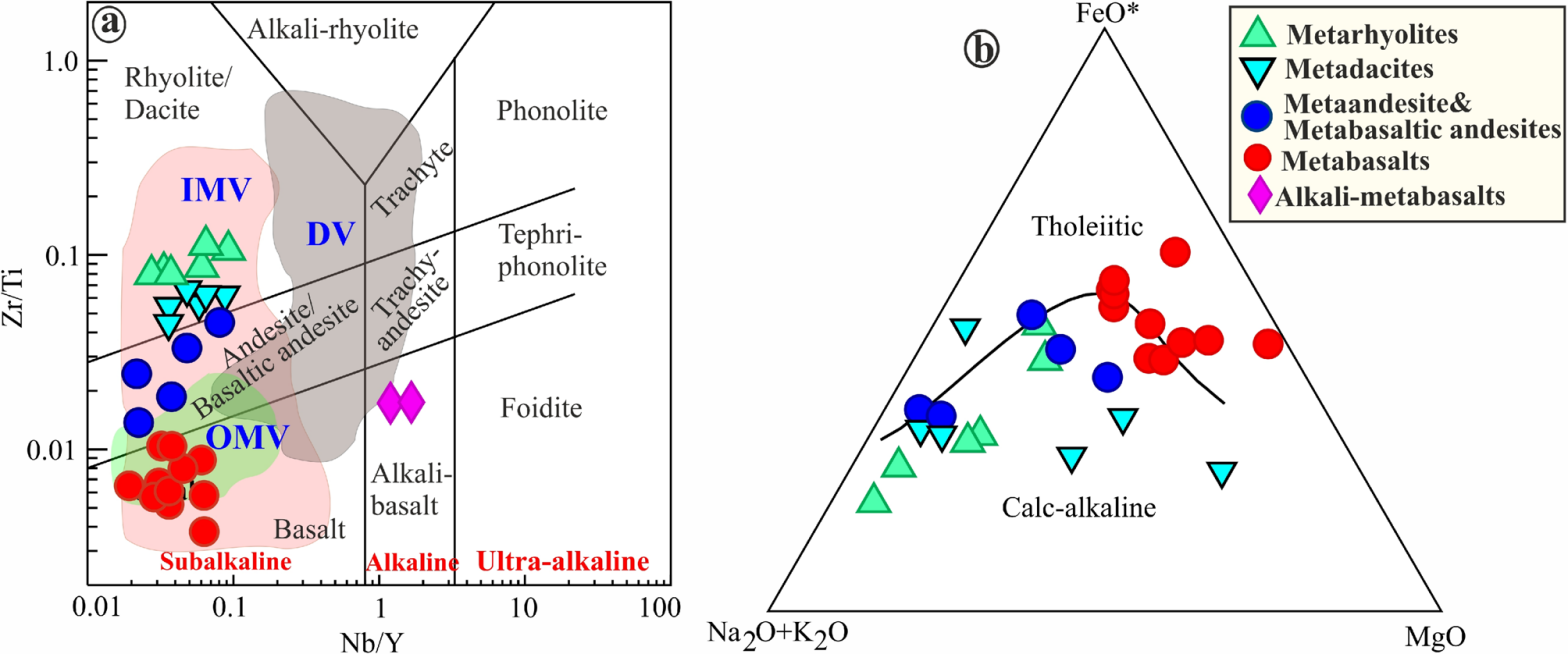
Whole-rock geochemistry of the WRAM. (**a**) Nb/Y vs. Zr/Ti classification diagram^73^ and (**b**) AFM discrimination diagram^78^. The fields of Dokhan volcanics (DV), the ophiolitic MV (OMV), and the island–arc MV (IMV) field after Khedr, et al.^43^. This figure is created by the Igpet 2010 software (https://www.rockware.com/product/igpet/).

Lineaments are simply linear or curvilinear edges that may be related to geologic structures (e.g., faults and joints), and/or linear valleys^63–66^. Satellite images are broadly used to extract lineaments for defining geologic structures and tectonic fabrics^67^. PC1 of Landsat-8 data carries most information (94.0%) (Supplementary Table 1b); therefore, it is suitable for lineament extraction in the studied WRAM. The process of lineament extraction was performed automatically using the Line module algorithm of the PCI Geomatica 2016 software. The extraction process relies significantly on various user-defined parameters. Key parameters in control include the Filter Radius, Curve Length Threshold pixel, Line Fitting Error Threshold, Angular Difference Threshold, Linking Distance Threshold, and Edge Gradient Threshold (Supplementary Fig. 1a). The results of lineament analysis and the density map of the investigated area reveal that metavolcanics, metagabbro, and granites are highly deformed and fractured (Supplementary Fig. 1b, c). Plotting lineaments on the rose diagram implies that structures in the study area have a random distribution and largely prevail along NE–SW and NW–SE directions, with rare distribution along E–W and N–S directions. The current NW–SE structural trend and its conjugated NE–SW and N–S trends crosscut the WRAM (Fig. 2) are consistent with the Najd fault system^68,69^, but the NE–SW lineaments are slightly longer and more abundant in number than those of other trends (Supplementary Fig. 1d). The Najd fault system (NW–SE) plays a critical role in controlling regional structural deformation in SED, acting as major shear zones that facilitated fluid flow and tectono-metamorphic processes aiding in localizing hydrothermal alteration zones and mineralization in the studied area (Figs. 3c–h, 4f, 5c- d), particularly for economic minerals (e.g. sulfides, copper and talc)^17,70^. The reliability of the remote sensing (RS) results is further validated by detailed field investigations (Fig. 3), as well as subsequent petrographic examinations (Figs. 6 and 7) and whole-rock geochemical data (Fig. 8a), which collectively support the interpreted lithological rock boundaries and alteration zones.

### Petrography

The Ranga metavolcanic rocks are predominantly composed of mafic and felsic varieties, with subordinate intermediate types (bimodal volcanics), as revealed by integrated remote sensing, detailed field investigations, and petrographic analyses. Mafic metavolcanics range in composition from metabasalts and alkali metabasalts grading into metabasaltic andesites (intermediate type) for some samples, whereas the lapilli metatuffs represent pyroclastic rocks. Metabasalts comprise plagioclase (45–65 vol.%) and mafic minerals (e.g., amphibole and a few clinopyroxene: Cpx, 15–30 vol.%) with discernible amounts of sulfides and Fe-Ti oxides (~ 10 vol.%). A few titanite, apatite, rutile, and quartz grains are recorded. These rocks exhibit both aphyric and porphyritic textures (Fig. 6a). Alkali metabasalts comprise mainly plagioclase (42–60 vol.%) and mafic minerals (e.g., Cpx and amphibole, 10–20 vol.%) with some opaque grains (< 10 vol.%), showing an intergranular texture (Fig. 6b). Plagioclase occurs as lath-shaped crystals (0.2–1.2 mm) with lamellar twinning, partly altered to sericite or clay minerals (e.g., kaolinite). The Cpx and amphibole appear as subhedral grains (0.1–1 mm) to prismatic crystals, occupying interstitial spaces. They are partially altered to chlorite or actinolite, whereas Cpx is mainly converted to amphibole.

Metabasaltic andesites comprise plagioclase (37‒60%) and mafic minerals such as amphibole (15‒25 vol.%) with subordinate quartz contents of ~ 5‒10 vol.% (Fig. 6c) and opaque minerals (< 8 vol.%). A few titanite, apatite, and rutile crystals are observed. Plagioclase occurs as microlaths (0.3–0.8 mm) and as tabular phenocrysts (1–2 mm) (Fig. 6a, c). Some plagioclase phenocrysts are partially to completely altered to kaolinite, sericite, and epidote (Fig. 6c). Amphiboles appear as prismatic and fibrous crystals (0.1–0.5 mm) and as fine groundmass (Fig. 6a, c). Lapilli tuffs are composed of rounded to elongated lithic andesite fragments (0.5–2 cm) with a subordinate amount of fine quartz set in a fine-grained groundmass (Fig. 6d). The intermediate samples are represented by subordinate metaandesites and are distinguished by relatively higher quartz content (10–15%) and lower amphibole content (10‒20%) compared to the metabasaltic andesite. Metaandesites consist mainly of plagioclase (50–65%) and mafic minerals such as amphibole, epidote, and chlorite with subordinate quartz and opaque minerals (e.g., sulfides and Fe-Ti oxides).

Felsic metavolcanics constitute chiefly metarhyolites and metadacites. Porphyritic metarhyolites are composed mainly of quartz (40‒55 vol.%) and plagioclase (20‒40 vol.%), and minor sulfides, Fe-Ti oxides, and mafic minerals (Fig. 6e). Rutile and apatite are accessory minerals. Porphyritic metadacites consist of quartz (35–45 vol.%) and plagioclase (20–45 vol.%) with subordinate mafic minerals and opaque minerals (up to 15 vol.%), which are mainly sulfide and a few Fe-Ti oxides. Quartz occurs as anhedral to subhedral phenocrysts with different grain sizes (1–2 mm). It is sometimes corroded by the groundmass and exhibits wavy extinction (Fig. 6e). Quartz in the groundmass is commonly fine-grained and sometimes intergrown with feldspars, forming a graphic texture. Plagioclase occurs as subhedral columnar crystals, twinned phenocrysts ranging in size from 0.5 to 1 mm long, and as fine anhedral grains in the groundmass (Fig. 6e). Some plagioclase crystals are completely altered to kaolinite, epidote, and sericite or saussurite (Fig. 6e, f). Minor chlorite and mica occur as small flakes and shreds embedded in the groundmass. Similarly, Atshan metavolcanics are of bimodal composition, having both mainly felsic and mafic varieties with minor metaandesite. The felsic varieties range in composition between metadacites and metarhyolites. In contrast, the mafic ones include metabasalts, alkali metabasalts, and metabasaltic andesites (Fig. 6f). Most metavolcanics are sheared, kaolinized, ferrigenated, and epidotized (Fig. 6e).

The different occurrences of talc-rich rocks along the shear zones include tremolite talc, silicified talc, and malachite-bearing talc varieties. The predominant talc rocks (bands and lenses) that are hosted in the metabasaltic andesite consist of > 70 vol.% talc with subordinate tremolite flakes, chlorite, and malachite (Fig. 6g). Tremolite appears as long fibrous or asbestiform crystals (2–3 mm), making up more than 60 vol.% of the tremolite talc rocks (Fig. 6g). Occasionally, the pure talc rocks grade into chlorite talc rocks with a relative abundance of chlorite and exhibit schistose texture. The minor talc lenses, which are hosted in the metabasalts, are composed mainly of talc with subordinate serpentine (Fig. 6h, i).

The examined polished thin sections of some mineralized metavolcanics indicate the existence of a distinct Fe–Cu–Zn sulfide mineralization. Sulfide mineralization occurs either as disseminated grains (Fig. 7a–e) or as massive types (Fig. 7g–h) within metavolcanics, jasperoid lenses, and Atshan tremolite talc rocks. Pyrite, chalcopyrite, and sphalerite are dominant Fe–Cu–Zn sulfide minerals, while chrysocolla is the only Si-bearing copper mineral. Pyrite crystals appear as creamy yellow subhedral cubic grains in the metavolcanic rocks (Fig. 7a–d), and a few altered grains exhibit a colloform texture (Fig. 7b). They are partially or completely replaced by goethite, especially within the jasperoid samples. Goethite occurs as grey-colored concentric rims around fresh pyrite cores and sometimes replaces the whole grain (Fig. 7b, e). Chalcopyrite and sphalerite commonly exist as small patches hosted in pyrite. Chalcopyrite is characterized by dark yellow, while sphalerite is light grey, with blebs intergrown with pyrite and chalcopyrite (Fig. 7f–h). Sphalerite appears as medium-grey grains intergrown with pyrite and chalcopyrite (Fig. 7g‒h). Green-colored chrysocolla veinlets are associated with talc in the Atshan tremolite talc samples (Fig. 7i).

### Whole-rock chemistry

Thirty representative samples of the WRAM were selected for major and trace-element analysis and are listed in Supplementary Table 2. These samples include 12 felsic, 5 intermediate to mafic, and 13 mafic metavolcanics. The WRAM generally exhibits low to moderate loss on ignition (LOI) values, which range from 0.77 to 4.69 wt.% in felsic rocks, 1.05 to 4.67 wt.% in intermediate to mafic varieties, and 0.9 to 3.15 wt.% in mafic rocks (Supplementary Table 2). In contrast, two mafic samples (Sr42 and Sr16) display markedly elevated LOI values of 8.8 wt.% and 10.3 wt.%, respectively (Supplementary Table 2), indicating localized intense alteration of primary plagioclase to secondary carbonate minerals. This interpretation is further supported by the low to slightly moderate alteration index^71,72^ (AI = 34% on average; Supplementary Table 2).

The current metavolcanics have a wide range of SiO_2_ contents (44‒77.23 wt.%; Supplementary Table 2) with a clear bimodal composition. The felsic metavolcanics are poor in TiO_2_ content (≤ 0.28 wt.%) relative to both the intermediate-mafic type (TiO_2_ < 0.79 wt.%) and typical mafic rocks (metabasalt; TiO_2_ < 0.89 wt.%) (Supplementary Table 2). In contrast, the alkali metabasalts have higher TiO₂ (TiO_2_, up to 3.4 wt.%) concentrations (Supplementary Table 2). Moreover, the Fe_2_O_3_ and MgO contents increase from the felsic (Fe_2_O_3_ < 3.56 and MgO < 4.98 wt.%) to the mafic types (Fe_2_O_3_ < 13.18 and MgO < 10.1 wt.%). The WRAM are chemically categorized into three main groups based on the Nb/Y vs. Zr/Ti diagram 73 (Fig. 8a): felsic, mafic, and subordinate intermediate to mafic rocks. The acidic rocks consist of metarhyolites and metadacites, while the mafic rocks are metabasalts and alkali metabasalts (Fig. 8a). The alkali metabasalts are represented by a group of six samples (Sr40–Sr42 and Sh17–Sh19) from the total collected samples, showing similar textures, mineral assemblages, and modal volume percentages; this is confirmed by petrography and SEM–EDS analyses. Due to the presence of multiple rock varieties in the study area, only two representative samples (Sr42 and Sh17; Supplementary Table 2) were selected for detailed whole-rock geochemical analysis. The intermediate to mafic rocks range in composition from metaandesites to metabasaltic andesites (Fig. 8a).

The WRAM resembles in composition the island arc metavolcanics (IMV; Fig. 8a)^16,18^, commonly referred to as Shadli metavolcanics in the CED and SED of Egypt. This inference is supported by plotting the studied samples in the IMV, distinguishing them from ophiolitic metavolcanics (OMV)^5,74^ and Dokhan volcanics (DV)^75–77^ (Fig. 8a). The Nb/Y vs. Zr/Ti diagram also reveals that the majority of WRAM rocks are predominantly subalkaline in composition; however, the alkali-metabasalts exhibit an alkaline affinity (Fig. 8a). SiO_2_–(Na_2_O + K_2_O) diagram^78^ also reflects subalkaline affinities for the majority of WRAM rocks, consistent with the classification based on the immobile-element Nb/Y–Zr/Ti diagram (Fig. 8a); however, the alkali-metabasalts display a scattered distribution between the alkaline and subalkaline fields (Supplementary Fig. 3a), likely reflecting minor modification of SiO_2_ and alkali contents during post-magmatic alteration. Therefore, the immobile-element diagram (Fig. 8a) considers a more reliable basis for the alkaline series. The AFM discrimination diagram^78^ distinguishes the subalkaline series into calc-alkaline and tholeiitic magma types (Fig. 8b). Almost all mafic rock samples have a tholeiitic composition compared with the calc-alkaline affinity of their counterparts of felsic to intermediate rocks (metarhyolites, metadacites, and metabasaltic andesites). Similarly, the subalkaline rocks show tholeiitic to calc-alkaline affinity based on the SiO_2_ vs. K_2_O diagram^79^, except one sample of metarhyolites plots within the high-K calc-alkaline field, indicating potassic enrichment (Supplementary Fig. 3b).

**Fig. 9:**
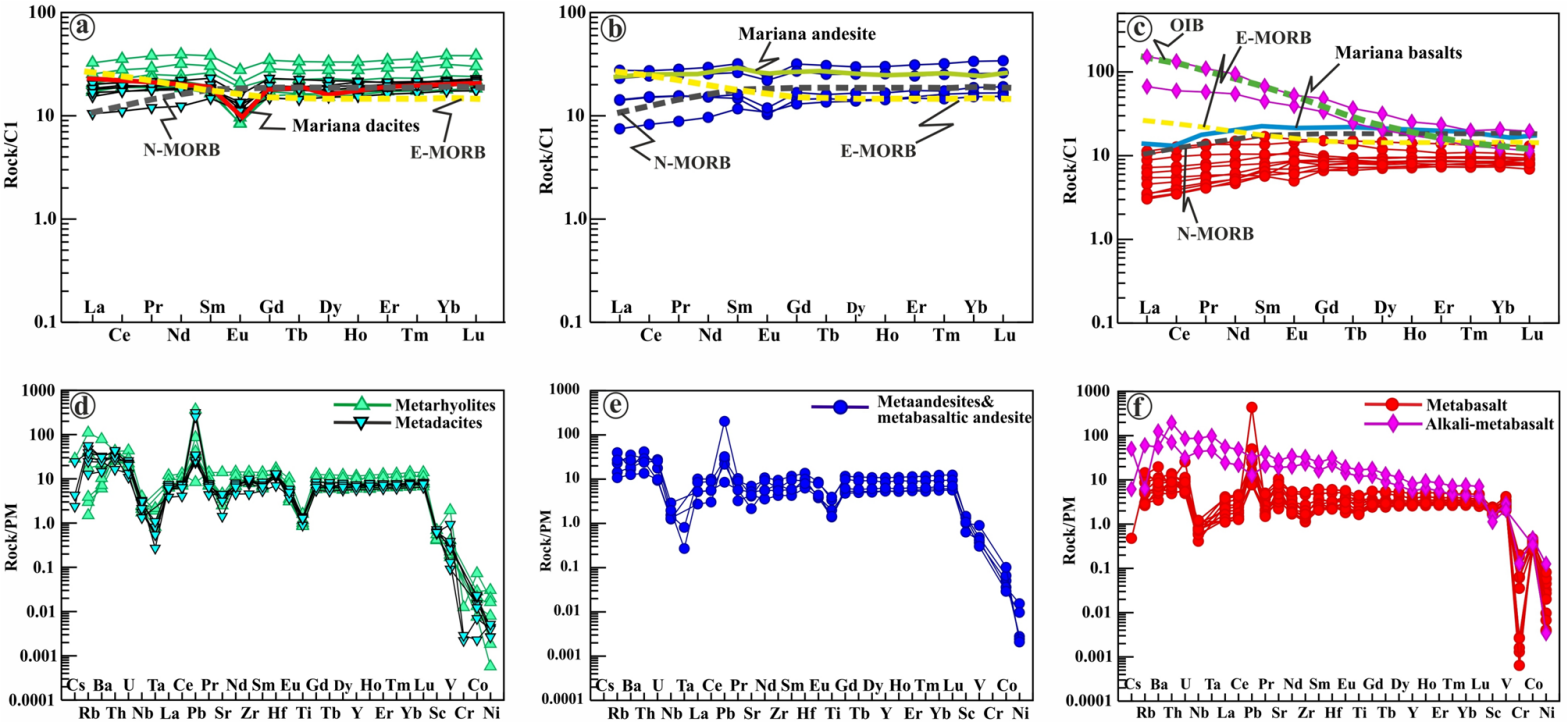
Trace element compositions of the WRAM. (**a**), (**b**), and (**c**) chondrite (C1)–normalized REE patterns^80^ of metarhyolites-metadacites, metaandesites-metabasaltic andesites, and metabasalts & alkali metabasalts; respectively, (**d**), (**e**), and (**f**) PM-normalized trace element patterns^80^ of metarhyolites-metadacites, metaandesites-metabasaltic andesites, metabasalts, and alkali metabasalts; respectively. The N-MORB, E-MORB, and OIB patterns are after Sun and McDonough^81^, while the Mariana rocks are after Pearce, et al.^82^. This figure is created by the Igpet 2010 software (https://www.rockware.com/product/igpet/).

The Wadi Ranga bimodal metavolcanics exhibit variations in the REE contents among the felsic, intermediate-mafic, and mafic rocks. The metarhyolites-metadacites and metaandesite-metabasaltic andesite are slightly higher in average REEs contents (ΣREEs = 48.1 and 52.7 ppm, respectively) than those of typical metabasalts (ΣREEs = 20 ppm on average; Supplementary Table 2). In contrast, the alkali metabasalts are highly rich in REE contents (up to 212 ppm; Supplementary Table 2) relative to all metavolcanics. The Chondrite (C1)-normalized REE patterns of the Ranga metarhyolites and metadacites (Fig. 9a) are nearly flat with nearly the same LREEs ((La/Sm)_N_ = 0.71‒1.08) as HREEs ((Gd/Yb)_N_ = 0.86‒1.04) and display a negative Eu anomaly ((Eu/Eu*)_N_ = 0.47–0.77; Supplementary Table 2). The metaandesites and metabasaltic andesites show flat REE patterns, where LREE contents resemble HREEs (Fig. 9b). However, these intermediate to mafic rocks are more depleted in both LREEs and HREEs ratios ((La/Sm)_N_ = 0.64‒0.89 and (Gd/Yb)_N_ = 0.84‒1.06) relative to the felsic types (Supplementary Table 2). Additionally, they display a less pronounced negative Eu anomaly ((Eu/Eu*)_N_ = 0.72–0.88; Supplementary Table 2) compared to the felsic rocks (Fig. 9b).

The chondrite-normalized REE pattern of the alkali metabasalts exhibits a steep trend, with significant enrichment in LREEs relative to HREEs ((La/Sm)_N_ = 1.5–2.25; Supplementary Table 2; Fig. 9c), in contrast to the typical metabasalts that display relatively flat HREEs ((Gd/Yb)_N_ = 0.84–1.41) with a slight LREE enrichment ((La/Sm)_N_ = 0.5–0.96). The alkali metabasalts show no significant Eu anomaly ((Eu/Eu*)_N_ = 0.92–1.02; Supplementary Table 2), compared with the pronounced negative Eu anomalies observed in other studied metavolcanics (Fig. 9c). The primitive mantle (PM)–normalized trace-element patterns^80^ of the studied metavolcanics are enriched in LILEs (Cs, Rb, Ba, Th, and U), relative to HFSEs such as Nb, Ta, Ti, and Zr (Fig. 9d–f). The alkali metabasalts are highly rich in LILEs, Nb (Nb > 28.9 ppm), and Ta (Ta > 1.7 ppm) contents compared to typical metabasalts (Nb < 2.6 and Ta < 0.08 ppm), but are distinguished by a notable negative Pb anomaly. Overall, the studied rocks have high average contents of base metals such as Cu (87.4 ppm), Zn (85.5 ppm), and Ni (37.8 ppm) (Supplementary Table 2). The notably high average Cr content (134.1 ppm) of the studied metavolcanics is attributed to the occurrence of ferromagnesian minerals, including pyroxene and amphibole.

### Mineral chemistry

#### Major oxides

**Fig. 10:**
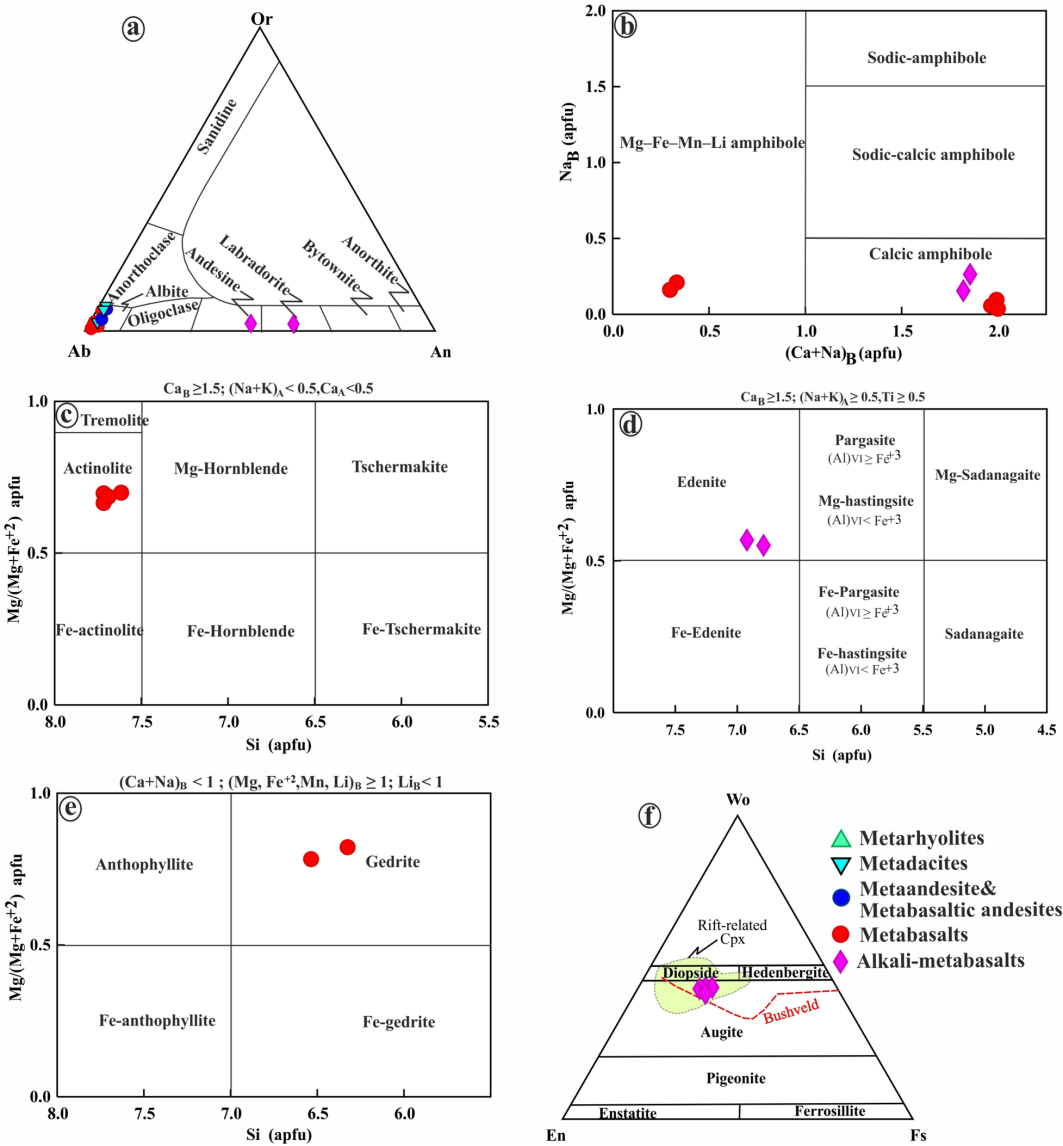
Mineral chemistry of plagioclase and amphibole minerals from the WRAM. (**a**) Ab‒An‒Or ternary classification diagram of plagioclase^83^, (**b**) (Ca + Na)_B_ vs Na_B_ classification diagram of amphibole types^84^, (**c**), (**d**) Si vs Mg/(Mg + Fe^2+^) classification diagrams of calcic amphiboles, respectively^84^, (**e**) Si vs. Mg/(Mg + Fe^2+^) classification diagrams of magnesian amphiboles^84^, (**f**) Enstatite (En)-Ferrosilite (Fs)-Wollastonite(Wo) ternary diagram of clinopyroxene^85^. The rift-related Cpx polygon is after Muravyeva, et al.^86^, Li, et al.^87^, and Zhu, et al.^88^; while the Bushveld line is after Atkins^89^. This figure is created by the Igpet 2010 software (https://www.rockware.com/product/igpet/).

The major-element contents of silicate minerals (plagioclase, amphibole, clinopyroxene, epidote, chlorite, and serpentine) in the WRAM and related talc rocks are determined by EPMA and listed in Supplementary Table 3a. Plagioclase is dominantly albite (An_0.94–10.34_), except in alkali metabasalts, where it ranges from andesine to labradorite (An_46.6–59.7_) (Supplementary Table 3a; Fig. 10a)^83^. Amphibole (Mg# = 0.41–0.8) in metabasalts and alkali metabasalts is classified into Ca-rich and Mg-rich varieties, including actinolite and edenite (Ca-amphiboles) and gedrite (Mg-amphibole) (Fig. 10b–e)^84^. The Cpx (Mg# = 0.66–0.69) in alkali metabasalts is augite in composition, plotting near the diopside–augite boundary^85^ with an average composition of Wo_43.2_-En_37.7_-Fs_19.2_. It includes a significant amount of Al_2_O_3_(4–5.25 wt.%), Na_2_O (~ 0.5 wt.%), Cr_2_O_3_ (up to 0.18 wt.%), and TiO_2_ (1.13‒2.06 wt.%), similar to primary Cpx in the rift setting^86–88^ and follows the Bushveld trend after Atkins^89^ (Fig. 10f). Epidote from metarhyolite, metadacite, and metabasalts comprises SiO_2_ (~ 38 wt.%) and CaO (23.1–23.7 wt.%) (Supplementary Table 3a), and falls into the epidote field (Supplementary Fig. 4a, b)^90^. Chlorite (Mg#, ~ 0.9) in Ranga-Atshan metadacites and rhyolites (Supplementary Table 3a) is talc chlorite in composition based on the Si vs. (Fe^2+^  + Fe^3+^) discrimination diagram (Supplementary Fig. 4c)^91^. It shows narrow ranges of Al_2_O_3_ (8.61–8.93 wt.%) and MgO (27.90–29.50 wt.%) (Supplementary Table 3a). Based on the Mg–Si–Fe^2+^ and SiO_2_–MgO discrimination diagrams^92,93^, the studied serpentines from Atshan talc rocks are antigorite (Supplementary Fig. 4d, e) with high SiO_2_ (42.7‒44.1 wt.%) and MgO (37.2‒38.1 wt.%) contents, resembling those of pseudomorphic serpentine recrystallized during prograde metamorphism (Supplementary Fig. 4f).

SEM–EDS analyses were conducted on sulfides, oxides, and alteration minerals (Supplementary Table 3b). Pyrite is the dominant sulfide phase, with Fe (45.4–46.5 wt.%) and S (49.8–54.7 wt.%) close to ideal FeS_2_ stoichiometry, with minor trace elements (Ni, Co, Ag < 0.5 wt.%). Chalcopyrite (CuFeS_2_) contains Cu (38.7–39.2 wt.%), Fe (28.1–28.5 wt.%), and S (~ 32 wt.%), while sphalerite is Zn-rich (Zn ~ 54.2 wt.%) with minor Fe (~ 9.8 wt.%), indicating Fe-rich sphalerite typical of higher-temperature hydrothermal conditions.

Oxide and hydroxide minerals include goethite (FeO; 79.1–79.5 wt.%), hematite (FeO; up to ~ 96 wt.%), and minor ilmenite (FeO ~ 46.8 wt.% and TiO_2_ ~ 49.2 wt.%). The close association of hematite with sulfides suggests oxidation of primary Fe-sulfides during hydrothermal alteration or supergene processes. Talc is characterized by high SiO_2_ (~ 60.3–65.1 wt.%) and MgO (~ 32.2–33.2 wt.%), consistent with Mg-rich alteration, whereas chrysocolla shows elevated CuO (~ 39.2–54.6 wt.%) and SiO_2_ (~ 48.1–52.6 wt.%), confirming its origin as a secondary Cu-silicate formed during supergene alteration. Additionally, silicates are analyzed, including oligoclase, actinolite, epidote, chlorite, and illite (Supplementary Table 3b); their data reflect greenschist-facies metamorphism and hydrothermal alteration under low- to moderate-temperature conditions.

#### In-situ trace elements

**Fig. 11:**
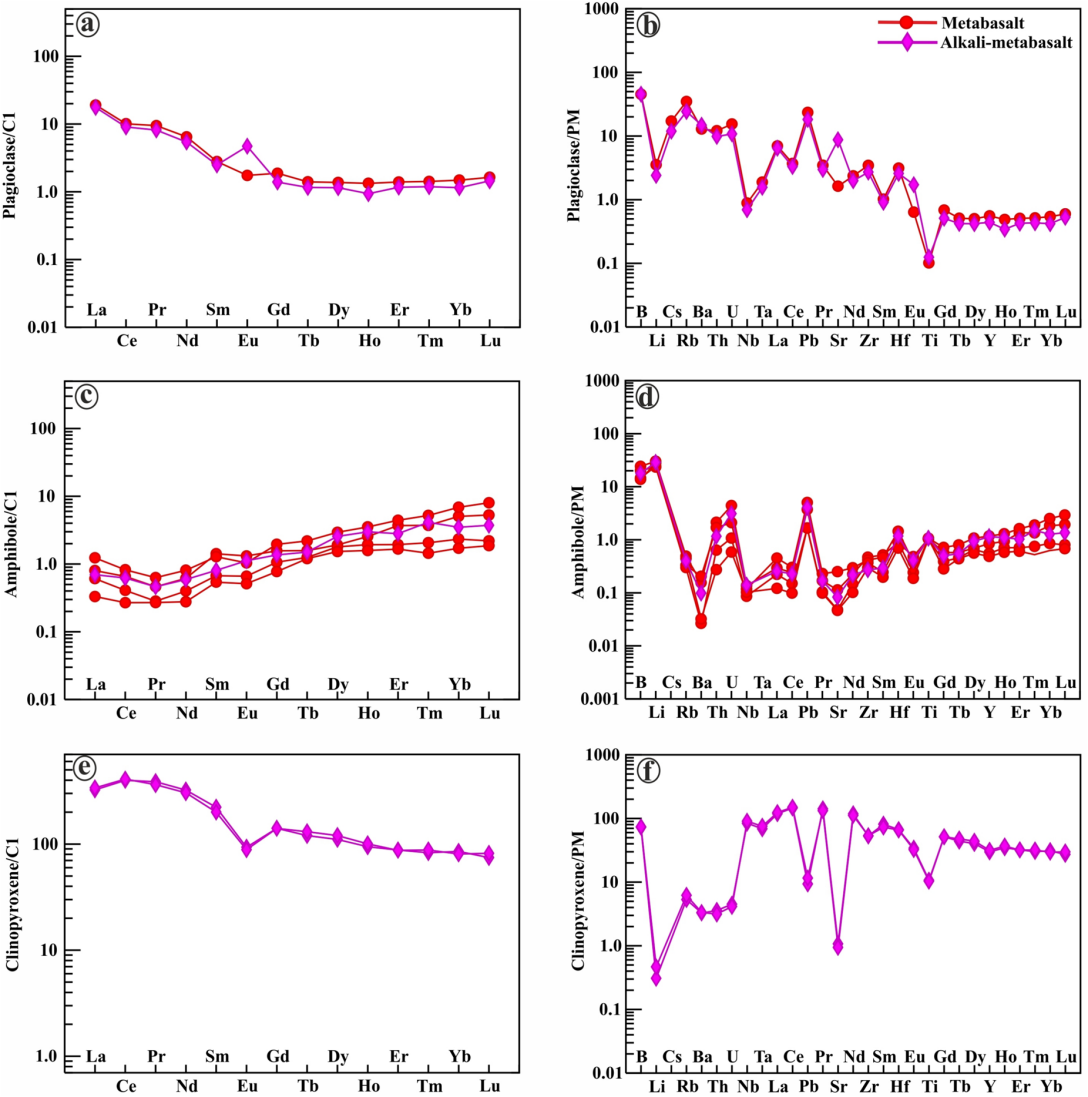
Chondrite (C1)-normalized REEs patterns and primitive mantle (PM)-normalized trace-element patterns of plagioclase (**a**), (**b**), amphiboles (**c**), (**d**), and clinopyroxene (**e**), (**f**). Normalized C1 and PM values are according to McDonough and Sun^80^. This figure is created by the Igpet 2010 software (https://www.rockware.com/product/igpet/).

The in-situ determination of trace and REE contents in plagioclase, amphibole, and epidote from the WRAM is listed in Supplementary Table 3c. Rare earth elements (REEs) of plagioclase (ΣREEs: 14.7–16.4) in both metabasalts and alkali metabasalts are normalized based on Chondrite (CI) values^80^. They exhibit slightly spoon-shaped patterns with enrichment of LREE compared with HREEs ((La/Lu)_N_ = 11.68–12.10; Fig. 11a). A pronounced positive Eu anomaly is observed in the alkali metabasalts (Eu/Eu* = 2.53, Fig. 11a), in contrast to the slightly negative Eu anomaly in metabasalts (Eu/Eu* = 0.76; Fig. 11a), suggesting more plagioclase accumulation in the former. The trace elements of plagioclase in alkali metabasalts are normalized according to primitive mantle (PM) values^80^. They are enriched in LILEs (e.g., Ba, Rb, U, Pb; Fig. 11b) and depleted in HFSEs (e.g., Nb, Ta, Ti; Fig. 11b). They also exhibit a more pronounced positive Sr anomaly compared to those of plagioclase in metabasalts (Sr/Sr* = 3.42 and 0.55, respectively; Supplementary Table 3c). Amphibole exhibits typical spoon-shaped patterns, where HREEs are higher in concentration than LREEs ((La/Lu)_N_ = 0.15–0.28; Fig. 11c) with a negative Eu anomaly (Eu/Eu* = 0.66–0.88) in metabasalts compared with a pronounced positive Eu anomaly (Eu/Eu* = 1.07; Fig. 11c) in alkali-metabasalts. The PM-normalized trace-element pattern of current amphibole is marked by enrichment in LILEs (Cs, Ba, and U; Fig. 11d), coupled with pronounced depletions in HFSEs (Nb, Ta, and Ti; Fig. 11d). The Cpx in alkali metabasalts displays an inclined dome or arc-shaped REE pattern with strong LREE enrichment relative to HREEs ((La/Lu)_N_ = 4.13–4.35; Fig. 11e and Supplementary Table 3c). A negative Eu anomaly (Eu/Eu* = 0.53; Supplementary Table 3c) appears due to the coexistence of Cpx with plagioclase at the same crystallization time. The PM-normalized trace-element patterns of the investigated Cpx are enriched in LILEs (e.g., Rb, Th, and U; Fig. 11f) and depleted in HFSEs (e.g., Nb, Ta, Ti; Fig. 11f), along with a pronounced negative Sr anomaly (Sr/Sr* = 0.01; Supplementary Table 3c).

## Discussion

### P–T condition of Wadi Ranga metavolcanics

**Fig. 12:**
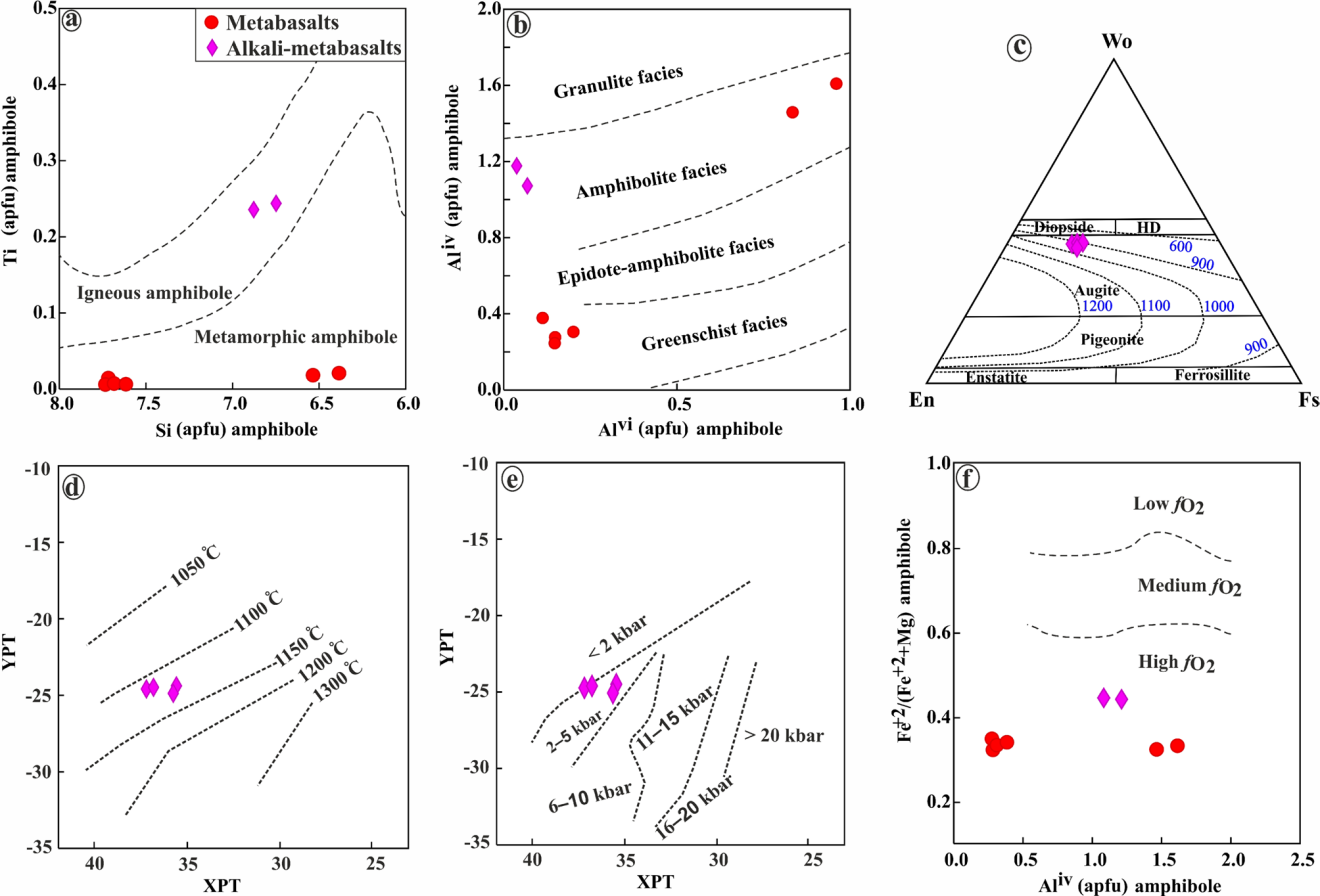
(**a**) Si vs. Ti discrimination diagram of amphiboles^84^, (**b**)Al^iv^ vs. Al^vi^ bivariate discrimination diagram of amphiboles^94^, (**c**) Wo-En-Fs nomenclature diagram of Cpx^85^. The isothermal dotted lines are after Stormer^95^, (**d, e**) XPT–YPT diagram for estimating formation temperature and pressure of Cpx^99^, (**f**) Al^iv^ vs. Fe^+2^/(Fe^+2^ + Mg^+2^) for oxygen fugacity estimation of amphiboles (*fO*_*2*_)^102^. This figure is created by the Igpet 2010 software (https://www.rockware.com/product/igpet/).

The mineral assemblages in the studied metavolcanic rocks reflect the low-grade regional metamorphism of the Ranga volcanic rocks. This is evidenced by the presence of secondary amphibole minerals, such as actinolite, edenite, and gedrite (Fig. 10c‒e), along with chlorite and epidote (Figs. 3g, 6d, and e). Furthermore, plagioclase within these rocks is partially to completely replaced by kaolinite, sericite, and saussurite (Fig. 6e and f). The majority of amphibole analyses plot within the metamorphic field (Fig. 12a), suggesting regional metamorphism (greenschist-amphibolite facies) rather than direct magmatic crystallization. This is consistent with the Al^iv^ vs. Al^vi^ bivariate diagram of Laird and Albee^94^, which shows that secondary amphibole minerals plot in the greenschist to amphibolite metamorphic facies (Fig. 12b). In contrast, amphiboles from the alkali metabasalts are rich in Ti and poor in Si contents, suggesting a primary igneous origin^84^ (Fig. 12a).

The majority of plagioclases and amphiboles have been significantly modified by metamorphic processes in the studied calc-alkaline to tholeiitic varieties; therefore, the estimation of P–T conditions is limited to the preserved primary amphibole and Cpx in alkali metabasalts (Supplementary Table 3a). The Cpx composition is predominantly augite and corresponds to equilibrium temperatures of approximately ~ 900–1000 °C (Fig. 12c)^95^; these estimated temperatures are slightly higher than thermometric calculations of amphibole^96,97^ temperature (~ 840–940 °C; Supplementary Table 3a). Based on primary pyroxene chemistry, the XPT–YPT equation and diagram^98,99^ are commonly used to estimate the formation pressure and temperature of the Cpx in alkali metabasalts (Fig. 12d, e). The calculated crystallization temperatures of Cpx range between 1100 and 1150 °C (Fig. 12d), whereas the estimated pressures fall within 2–5 kbar (Fig. 12e). This temperature is consistent with primary amphibole chemistry that was used to estimate temperature (1000–1070 °C; Supplementary Table 3a) based on the thermometric equation of Putirka^100^. In addition, the estimated pressure suggests a narrow range of ~ 1.9–2.8 kbar based on XPT–YPT diagram of Cpx (Fig. 12e) and estimated pressure using Supplementary Table 3a^101^. The oxygen fugacity (ƒO_2_) of both metabasalts is estimated based on the relationship between the Al^iv^ and Fe^2+^/(Fe^2+^ + Mg) ratio of amphiboles, which suggests their formation under high oxygen fugacity (Fig. 12f)^102^ due to slab-derived fluids in the subduction zone during the generation of volcanic protoliths of the studied metavolcanics.

### Petrogenesis of metavolcanics

#### Nature and source of magmas

Based on field observations, petrography, remote sensing, and geochemical data (Figs. 3, 4, 5, 6, 7 and 8), the WRAM are mainly classified into felsic and mafic varieties with subordinate intermediate to mafic types. The wide variations of the metavolcanics from felsic (metarhyolites and metadacites) to mafic (metabasalts), along with minor intermediate (metaandesites) (Fig. 8a), suggest the bimodal type (bimodal volcanism) and variations in compositions of parent magmas. The majority of the Wadi Ranga metavolcanics are classified as subalkaline metavolcanics, either tholeiitic or calc-alkaline (Fig. 8b)^78^, which can be distinguished by the AFM diagram (Fig. 8c). The AFM and SiO_2_ vs. K_2_O discrimination diagrams of Irvine and Baragar^78^ indicate that the WRAM exhibit both tholeiitic and calc-alkaline affinities (Fig. 8b and Supplementary Fig. 3b), consistent with magma generation in a supra-subduction zone (SSZ) environment. The mafic varieties are characterized by tholeiitic trends, whereas the felsic and intermediate compositions exhibit a predominantly calc-alkaline signature (typical for arc-related settings). The tholeiitic magmas as parent melts of the studied basalts are possibly generated during mantle plume upwelling (Fig. 8b), where tholeiitic to calc-alkaline magmas are characterized by mantle-derived magmas generated in the Supra-subduction zone (SSZ)^103^. The SiO₂–K₂O diagram^79^ further supports tholeiitic, calc-alkaline, and high-K calc-alkaline affinities (Supplementary Fig. 3b). The mafic samples almost occupy the tholeiitic field, while intermediate or intermediate to mafic ones (e.g., basaltic metaandesites) have tholeiitic to calc-alkaline affinities.

**Fig. 13:**
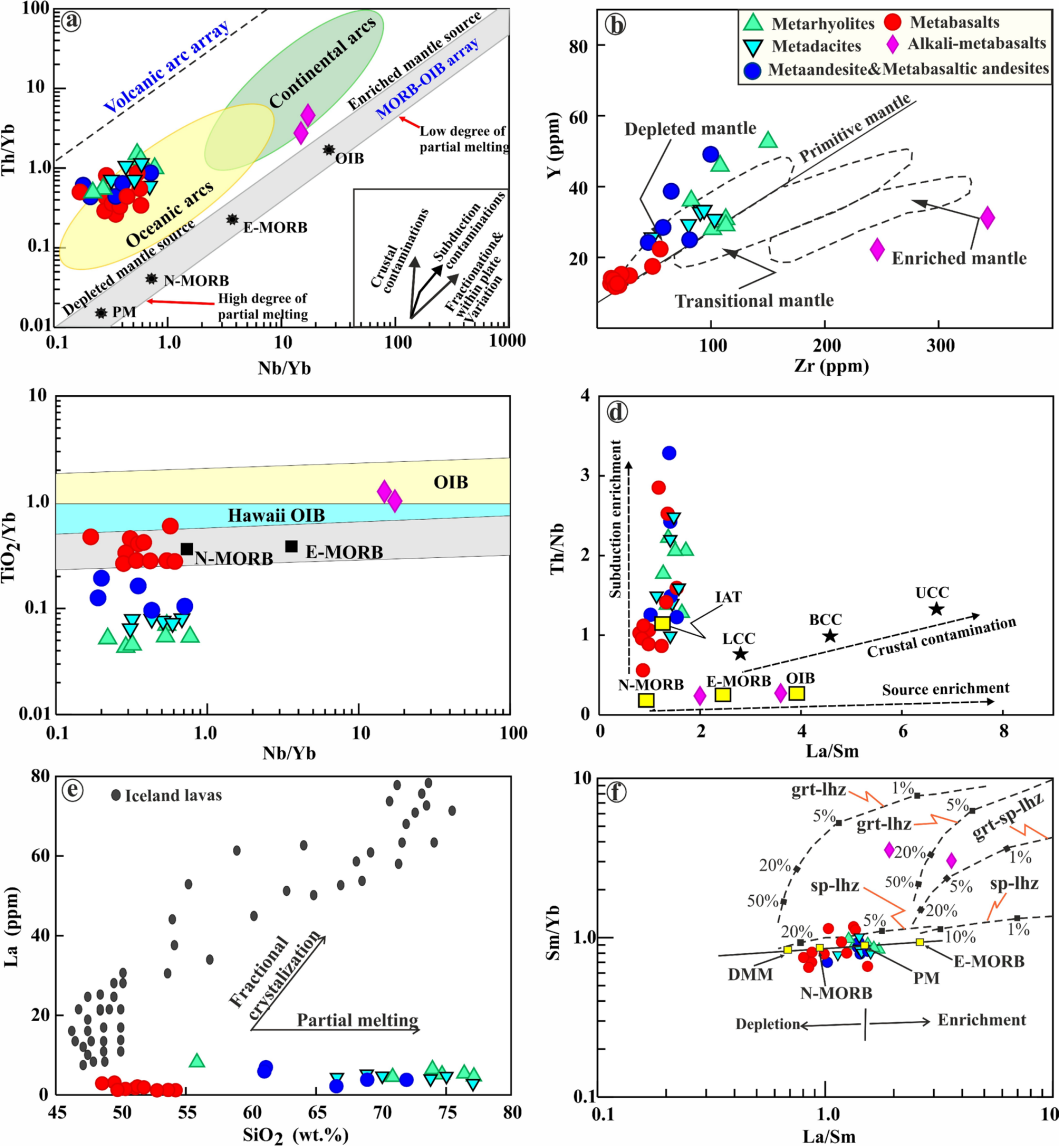
Whole-rock chemistry shows magma type and source of the WRAM. (**a**) Nb/Yb vs. Th/Yb^104^, (**b**) Zr vs. Y diagram^105^, (**c**) TiO₂/Yb vs. Nb/Yb diagram^106^, (**d**) Th/Nb vs. La/Sm diagram^109^, (**e**) SiO_2_ vs. La diagram modified after Brophy^110^, (**f**) La/Sm vs. Sm/Yb diagram^111^. Abbreviation: primitive mantle (PM), depleted MORB mantle (DMM), normal mid-ocean ridge basalts (N–MORB), enriched mid-ocean ridge basalts (E–MORB), ocean island basalt (OIB), garnet (grt), spinel (sp), lherzolite (lhz), and upper continental crust (UCC). PM, DMM, N–MORB, E–MORB, and OIB compositions are after Sun and McDonough^81^, the data of the Iceland lavas are after Brophy^110^, and the UCC composition is after Taylor and McLennan^112^. This figure is created by the Igpet 2010 software (https://www.rockware.com/product/igpet/).

Almost all of the Wadi Ranga metavolcanics have low (Nb/Yb) and (Th/Yb) ratios (Supplementary Table 2; < 0.77 and < 1.11, respectively) and plot within or near the oceanic-arc field (Fig. 13a)^104^, indicating derivation of their protoliths from a depleted-mantle source. In contrast, the alkali metabasalts have high (Nb/Yb) and (Th/Yb) ratios (Supplementary Table 2; > 14.8 and > 2.88, respectively) and plot within the continental arc generated from an enriched mantle source, like OIB magmas (Fig. 13a and b). This suggests involvement of an enriched mantle source and a lower degree of partial melting during the generation of parental magmas of Wadi Ranga alkali metabasalts (Fig. 13a)^104^. This interpretation is further supported by the Zr–Y diagram (Fig. 13b)^105^, where all the studied metavolcanic plots in the depleted mantle field, except alkali metabasalts, plot distinctly in the enriched mantle field. Additionally, by using the TiO_2_/Yb vs. Nb/Yb diagram (Fig. 13c)^106^, the alkali metabasalts plot in the space of OIB, supporting their derivation of their protoliths (e.g., alkali basalts) from an enriched mantle source.

In contrast, the metarhyolites and metadacite, as well as metaandesites and metabasalts, plot within the MORB array and close to the N-MORB field; this is consistent with a depleted mantle source for the majority of the Wadi Ranga volcanic rocks (Fig. 13c)^106^. Therefore, alkali basalts crystallized from fertile magmas from an enriched mantle source, which occurs as a spot or a narrow region surrounded by the depleted mantle; this is probably evidence of a mantle plume and mantle plume-driven magmatism^107,108^. The depleted mantle is the main source for most volcanic rocks, except alkali basalt, in the Wadi Ranga-Atshan area.

The two representative alkali metabasalts samples (Supplementary Table 2) have Ni (245.1 ppm) and Cr (337.8 ppm) contents consistent with ~ 70% of plume basalts that typically exhibit Ni > 150 ppm and Cr > 300 ppm^113^. Not only these elements, but also the higher HFSEs (Nb ~ 57.5 ppm, Ta ~ 3.6, Zr ~ 342, Hf ~ 9, Ti ~ 20,306) contents and Nb/U (33‒45) as well as Ce/Pb (17‒19) ratios for these alkali metabasalts than other types are the strongest indicator of plume-related melts (e.g., plume-derived basalt) for the generation of these rock photoliths. In contrast, non-plume magmatism (MORBs and Arc basalts) generally displays lower concentrations of HFSE, Ni, Cr, Nb/U and Ce/Pb. In addition, the chondrite-normalized REEs patterns of metarhyolites, metadacites, metaandesites-metabasaltic andesite, and metabasalts in the Wadi Ranga resemble N-MORB^81^, which is characterized by flat to slightly enriched LREEs and relatively flat HREEs (Fig. 9a‒c). This indicates the parent magmas of the studied metavolcanic protoliths formed from a depleted mantle source, except alkali basalts derived from melts generated from the enriched mantle spot during plume interaction. The enriched mantle source of our alkali basalts is supported by the strong LREE enrichment and a flat to slightly declining HREE pattern (Fig. 9c), closely resembling OIB^81^.

The increase of average REEs from mafic (metabasalts with ΣREEs: 20 ppm) through intermediate (ΣREEs: 48.1 ppm) to felsic rocks such as metarhyolites and metadacites (ΣREEs: 52.7 ppm; Supplementary Table 2) suggests that the protoliths of these metavolcanic rocks fractionally crystallized from the same tholeiitic basaltic magmas, whereas tholeiitic basalts crystallized first, and then andesite and felsic types (dacite then rhyolite) formed during magmatic fractionation. Moreover, the significant negative anomalies of Ti and Eu in the felsic rocks suggest fractionation of Ti-oxides and Ca-rich plagioclase, respectively, during the first crystallization of basalts^87,114^. The effect of fractional crystallization is evidenced through the positive correlation observed in the variation diagrams of Zr against some of the REEs and trace elements (Y, Yb, Nb, and Th) (Supplementary Fig. 5). On the other hand, the alkali metabasalts are enriched in REEs (ΣREEs: up to 212 ppm; Supplementary Table 2) relative to other tholeiitic to calc-alkaline metavolcanics, reflecting the derivation of alkali basalts from a more enriched mantle source^108^, like mantle plume-derived magmas or mantle plume interaction with the lithosphere.

Although felsic magmas may originate from partial melting of the crust, the geochemical characteristics of the studied metavolcanics do not support significant crustal involvement. The LILE/HFSE ratios, commonly used to assess crustal contribution^115,116^, show that the (La/Yb), (La/Sm), and (Th/Hf) values of the studied rocks (0.55–1.6, 0.8–1.7, and 0.4–1.5, respectively; Supplementary Table 2) are significantly lower than those of the upper continental crust (15.8, 6.6, and 1.9, respectively)^116^. Similarly, (La/Yb), (La/Sm), and (Th/Hf) ratios of alkali metabasalts (8–10.9, 2.4–3.6, and 0.9–1.8, respectively; Supplementary Table 2) are lower than those of the upper continental crust (La/Yb: ~ 15.8, La/Sm: ~ 6.6, and Th/Hf: ~ 2)^116^. Furthermore, the Th/Nb vs. La/Sm diagram modified after Piercey, et al.^117^ further supports negligible crustal contamination, as all studied samples plot away from the crustal contamination trend (Fig. 13d). Moreover, this aligns closely with the subduction–related enrichment trend, showing compositions that cluster around N-MORB^81^ and island arc tholeiite (IAT) fields (Fig. 13d). In contrast, the alkali metabasalts plot within the E-MORB to OIB field^81^, suggesting generation of alkali basalts from an enriched mantle source, like mantle-plume-derived magmas or intermixed magmas generated during interaction of the base of the lithosphere with the head of the mantle plume (Fig. 13d)^103,118^. This is consistent with results of Stern, et al.^20^ for the same metavolcanic samples (Supplementary Table 2), whereas low‑δ^18^O of zircon (4.4–4.6 ‰) and its εHf(t) (~ + 10 to + 18), and whole‑rock initial ^87^Sr/^86^Sr (0.70206–0.70330) and εNd₍₇₃₉₎ (~ + 0.4 to + 8.6) indicate variably depleted, juvenile mantle sources with negligible input from upper continental crust. Likewise, the magma immiscibility model for felsic magma formation typically produces compositional gaps (i.e., absence of intermediate compositions and non-continuous or negatively correlated trends between mafic and felsic rocks), along with diagnostic petrographic features such as melt globules or droplets. The absence of these features in the studied samples argues against magma immiscibility as a viable process. Accordingly, fractional crystallization from a common mantle-derived tholeiitic parent magma is considered the most plausible mechanism for the generation of the felsic metavolcanics, whereas crustal melting and magma immiscibility are not supported by the available geochemical, isotopic, and petrographic evidence.

REE signatures of the parental melts in equilibrium with clinopyroxene from the alkali metabasalts were reconstructed using Cpx–melt partition coefficients^119,120^ (Supplementary Fig. 6 and Supplementary Table 3d). The chondrite-normalized REE patterns of these equilibrium parental melts show strongly fractionated trends with marked LREE enrichment over HREE, comparable to those of ocean-island basalt melts OIB^81^. This REE behavior reflects an enriched mantle source and supports magma generation under rift-related intraplate conditions, consistent with mantle plume involvement. The negative correlation between La and SiO₂ (Fig. 13e) supports that the mafic magma originated from partial melting of a MORB-like mantle source, later evolving into felsic compositions through fractional crystallization. The La/Sm (0.8–1.7) and Sm/Yb (0.65–1.2) ratios of the studied metavolcanics are lower than those of the garnet-lherzolite and consistent with their generation by 5–20% partial melting of a spinel-lherzolite mantle source (Fig. 13f)^111^. The studied metavolcanics resemble in composition Hamamid and Darhib volcanics^16,17^, which were generated by 10–15% and 5–20% partial melting of a spinel-lherzolite source, respectively. In contrast, the alkali basalt most likely originated from low-degree partial melting (5%) of a garnet lherzolite or garnet-spinel lherzolite mantle source (Fig. 13f)^111^ or from mantle plume-derived melts in the deeper mantle. This plume may interact with the base of the lithosphere, triggering mantle-derived melts that are intermixed with plume head-derived melts (like E-MORB or OIB). This is consistent with global observations that alkali basalts often crystallize from more enriched mantle sources (e.g., E-MORB or OIB) compared to tholeiitic basalts^121^. The different partial melting, magmatic affinity, and magma source (mixed plume–arc magmas) reflect a heterogeneous mantle source beneath the ANS.

#### Tectonic setting implications

**Fig. 14:**
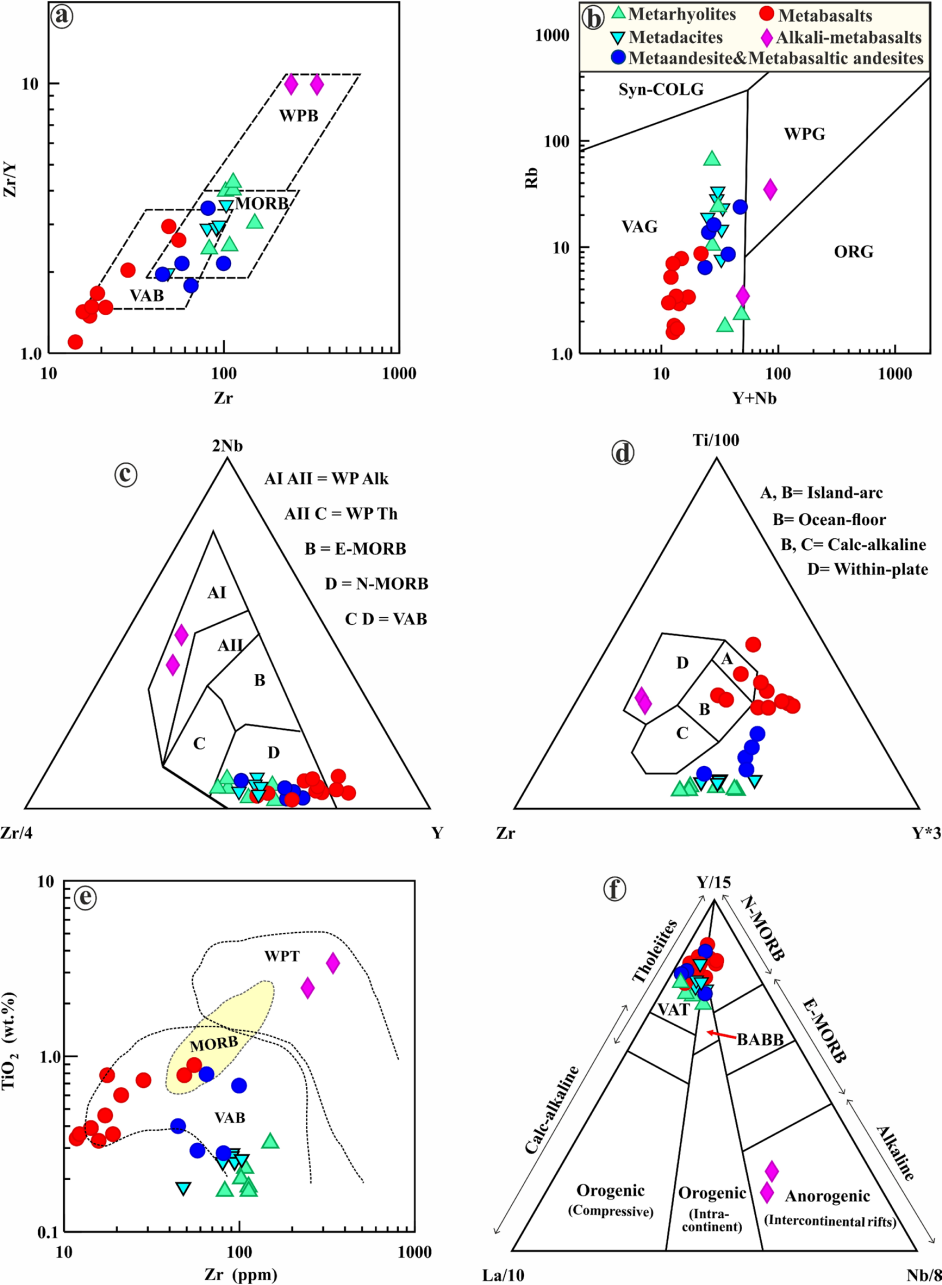
Whole-rock chemistry showing tectonic settings of the WRAM. (**a**) Zr vs. Zr/Y discrimination diagram^122^, (**b**) (Y + Nb) vs. Rb discrimination diagram^123^, (**c**) Zr/4–2Nb–Y ternary diagram^126^, (**d**) Y*3‒Zr‒Ti/100 ternary diagram^127^, (**e**) TiO₂ vs. Zr diagram^122^, (**f**) La/10‒Y/15‒Nb/8 ternary diagram^128^. Abbreviations: syn-collisional granites (Syn-COLG), within-plate granites (WPG), volcanic arc granites (VAG), ocean ridge granites (ORG), within-plate basalts (WPB), within-plate alkaline (WP.ALK), within-plate tholeiites (WP.Th), ocean island basalts (OIB), calc-alkaline basalts (CAB), island arc tholeiites (IAT), volcanic arc basalts (VAB), back-arc basin basalts (BABB), mid-ocean ridge basalts (MORB), enriched mid-ocean ridge basalts (E-MORB), normal mid-ocean ridge basalts (N-MORB). This figure is created by the Igpet 2010 software (https://www.rockware.com/product/igpet/).

**Fig. 15:**
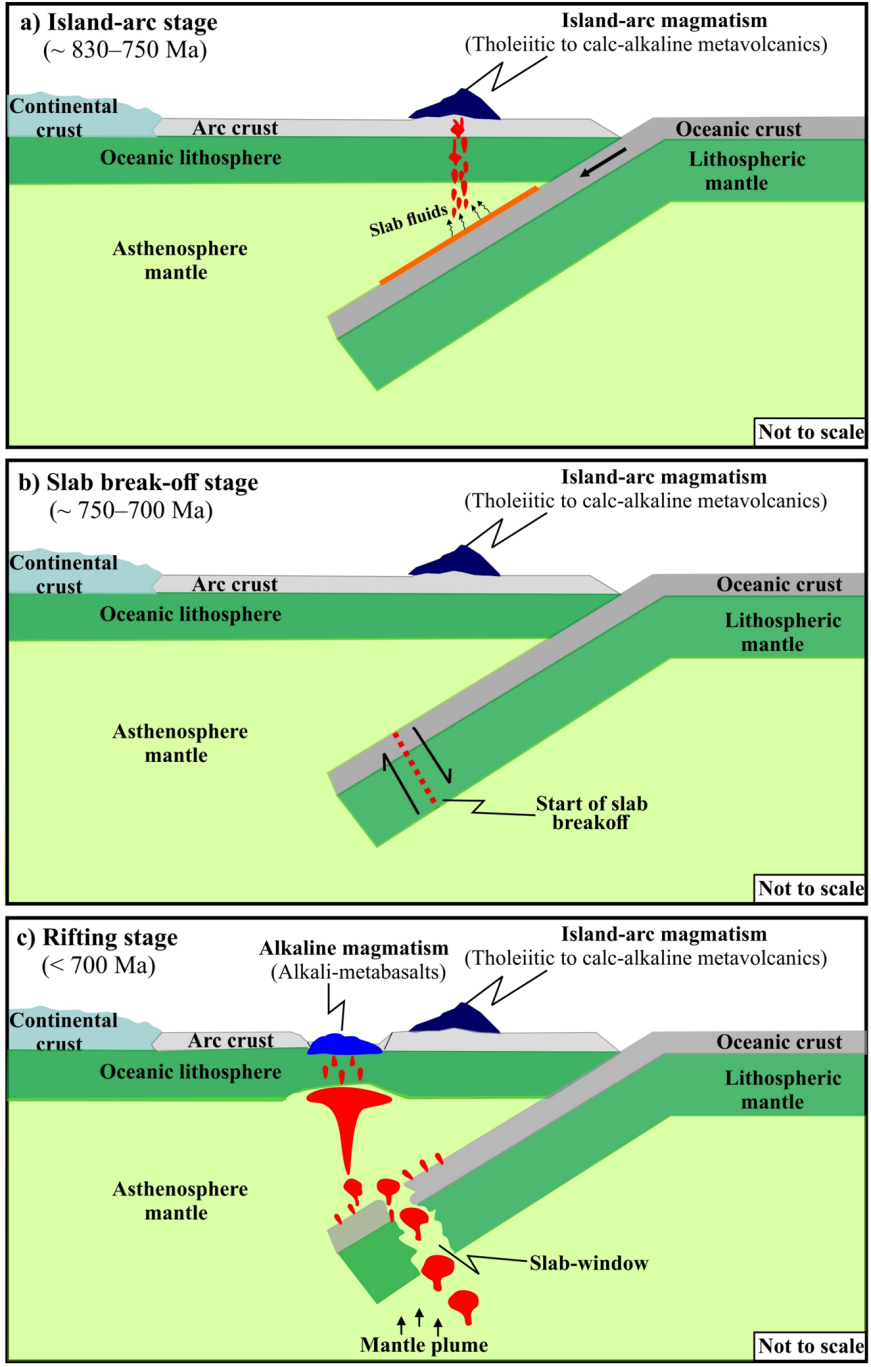
Simplified tectonic sketch of the WRAM in a subduction–rifting setting. (**a**) arc-stage: arc magmatism driven by slab-derived fluids, (**b**) Slab-break-off stage: detachment of the subducting oceanic lithosphere reduces slab-fluid input and initiates mantle upwelling, (**c**) rifting stage: opening of a slab window and mantle plume upwelling generate alkaline magmatism. Geochronological ages are after Gamal El Dien, et al.^19^. This sketch is drawn using CorelDRAW 2017 software (https://www.coreldraw.com/en/).

The Wadi Ranga bimodal metavolcanics, comprising various rock types ranging from metarhyolites to metabasalts, indicate a complex magmatic evolution that reflects a significant tectonic transition from an early subduction-related setting to a later intraplate, mantle plume-influenced regime. Geochemical discrimination diagrams support a supra-subduction zone (SSZ) environment for the earlier magmatic phase (Fig. 14). The tholeiitic and calc-alkaline metavolcanics mainly plot within the volcanic-arc basalt (VAB) and MORB fields, where alkali metabasalts fall within the within-plate basalt field based on the Zr vs. Zr/Yb diagram^122^ (Fig. 14a), consistent with magmas generated in the arc setting followed by within-plate rifting (Fig. 15). Furthermore, the Rb vs. (Y + Nb) diagram^123^ confirms the volcanic-arc origin of the tholeiitic and calc-alkaline metavolcanics, where they plot mainly within the volcanic-arc granite (VAG) field (Fig. 14b), supporting their derivation in a compressional supra-subduction zone (SSZ) setting. In contrast, only one alkali-metabasalt sample exists within the within-plate granite (WPG) field (Fig. 14b), likely due to Rb mobility during alteration. This agrees with the REE patterns of the studied metarhyolites to metabasalts that closely resemble those of Mariana arc magmas^82,124^, which have subduction-related characteristics with enrichments in LREEs over HREEs (Fig. 9a–c).

The occurrence of basaltic pillow lava structure (Fig. 3b) combined with the arc-related geochemical signatures is consistent with formation in a subduction-related environment (e.g., an island-arc setting) of the tholeiitic to calc-alkaline rocks^125^. The Y‒Zr/4‒2Nb ternary diagram shows that the majority of analyzed samples fall within the N-MORB and volcanic arc basalts (VAB) fields- related to the SSZ setting, except alkali metabasalts fall in the within-plate alkaline field (Fig. 14c)^126^. In addition, based on the Y*3‒Zr‒Ti/100 ternary diagram classification^127^ (Fig. 14d), all WRAM plot mainly in the island-arc and ocean floor fields, and others outside the definite fields, except alkali metabasalts plot in the within-plate field. The TiO₂ vs. Zr diagram further confirms the arc affinity, where the tholeiitic and calc-alkaline rocks plot predominantly within the volcanic arc basalts (VAB), while the alkali metabasalts fall in the within-plate tholeiite (WPT) field (Fig. 13e)^122^. Cabanis^128^ discriminated between anorogenic intercontinental rift, orogenic intracontinental, and orogenic compressive regimes based on the La/10–Y/15–Nb/8 ternary diagram.

The studied metavolcanics plot in the volcanic arc tholeiite (VAT) of a compressive setting (like a subduction-related setting), except the alkali metabasalts fall within the anorogenic (intercontinental rift) field (Fig. 14f); this is evidence of the extensional, plume-related intra-rift magmatism overprinting the earlier subduction regime. This interpretation is further supported by clinopyroxene compositions (Fig. 10f), where the alkali metabasalts fall within the augite field, overlapping with the typical domain of rift-related clinopyroxenes^86–88^. Finally, the chemistry of Cpx is widely used as a robust indicator of tectonic setting. Accordingly, the analyzed Cpx from the alkali metabasalts is plotted on the F1–F2 discrimination diagram^129^, which shows that most Cpx compositions cluster within the Within-Plate Tholeiitic (WPT) and Within-Plate Alkaline (WPA) fields (Supplementary Fig. 7). This distribution indicates that the parental magmas of the alkali metabasalts were predominantly derived from an intraplate mantle source.

### Geodynamic evolution of WRAM metavolcanics: evidence for a subduction–rift transition

The geodynamic evolution of the WRAM reflects a progressive transition from subduction-related arc magmatism to plume-influenced rifting, as constrained by integrated geochemical data (Fig. 15). This evolution is interpreted through a three-stage model, in which each stage is supported by diagnostic geochemical signatures.

During the arc stage (Fig. 15a), oceanic subduction dominated the tectonic configuration, with the downgoing oceanic slab releasing aqueous fluids into the overlying mantle wedge. This process induced partial melting, producing tholeiitic to calc-alkaline magmas enriched in water and depleted in HFSE (e.g., Nb, Ta). This stage is supported by the typical arc geochemical signatures, including enrichment in LILE and depletion in HFSE of the tholeiitic to calc-alkaline rocks (e.g., Nb and Ta; Fig. 9); these chemical signatures reflect dehydration-driven flux melting in a subduction zone setting. Mineral chemistry of actinolite and gedrite further supports this interpretation and indicates the hydrous condition of subduction-related settings.

This was followed by a slab break-off stage (Fig. 15b), interpreted to reflect the detachment of the subducting oceanic lithosphere, likely driven by changes in plate kinematics or collisional processes. This process facilitated asthenospheric upwelling and the development of a slab window, which disrupted the subduction-related magmatic flux and generated transitional volcanic compositions that retain arc geochemical signatures while showing incipient intraplate affinities. Such features are consistent with a geodynamic shift from compressional to extensional regimes, marking a major tectonic reorganization. There are indirect lines of evidence for slab break-off in this study. For instance, the MORB–OIB mixing trends, coexistence of Nb–Ta depletion with HFSE enrichment, transitional geochemical signatures, and transitional volcanic compositions from arc geochemical signatures to incipient intraplate affinities reflect slab-window opening and the upwelling mantle plume. Such hybrid arc and intraplate signatures are commonly interpreted in many tectonic models as evidence for upwelling plumes through slab-window opening during the slab break-off.

The rift stage (Fig. 15c) is represented by the emplacement of alkali metabasalts with clear within-plate geochemical signatures (high Nb, Ta, Zr, and Ti; Supplementary Table 2), WPB/OIB-like affinities (Fig. 14), and rift-related clinopyroxene compositions (Fig. 10f). The occurrence of this clinopyroxene and Ti–rich amphiboles such as edenite (Fig. 10d) is consistent with intraplate or rift-related magmas. This edenite crystallized from alkali- and Ti–rich magmas (TiO_2_ ~ 2 wt.%; Supplementary Table 3a), consistent with intraplate or rift-related settings^130^. This contrasts with the actinolite and gedrite compositions observed in the calc-alkaline to tholeiitic group, which are typical of subduction-related environments^84^. On the other hand, clinopyroxene-equilibrated melts from alkali metabasalts (Supplementary Fig. 6), which resemble OIB and take their arrays^81^, may represent the evolved rift stage. Alkali metabasalts are interpreted as products of mantle-plume-related magmatism, generated by partial melting of an enriched mantle source and/or mixing between plume-derived melts and depleted mantle components. The development of these alkaline magmas reflects an extensional tectonic regime and elevated thermal gradients, likely associated with mantle plume upwelling beneath the thinned lithosphere.

The mantle plumes may form a spot or region of enriched mantle within the depleted mantle above the subducting slab. This process has been documented in the Neoproterozoic metavolcanics of Kolet Um Kharit in the SED Desert of Egypt, which exhibit a mixing trend between depleted MORB-like and enriched OIB-like mantle sources^131^, interpreted as evidence for plume involvement^132^. The strong Nb and Ta depletion observed in both Kolet Um Kharit and the present metavolcanics may be attributed to their parent magmas derived from a depleted mantle source in the SSZ setting. Teklay, et al.^133^ similarly reported that the Nakfa arc metavolcanics in Eritrea (ANS) lie along the MORB–OIB array and originated from an enriched mantle source, supporting a plume-influenced arc magmatism model. The studied WRAM provides compelling evidence for such a transition, capturing the interplay between subduction dynamics and plume-related processes during the tectonic evolution of the ANS.

Stern, et al.^20^ reported positive εNd(t) values (~ + 0.4 to + 8.6), low ^87^Sr/^86^Sr ratios (~ 0.70206–0.70330), and low zircon δ^18^O values (~ 4.4–4.6‰), indicating a juvenile depleted mantle. The slight variability in εNd(t) and Nb–Ta depletion reflects modification by slab-derived fluids. These isotopic data indicate that the studied WRAM is interpreted as a mantle-plume–related hotspot developed beneath the ANS. On the other hand, the plume involvement is inferred from the coexistence of arc- and OIB-like signatures, HFSE enrichment (e.g., Nb, Ta, Zr, Ti), and MORB–OIB mixing trends (Figs. 9, 13, and 14). The studied area is part of the Shadli-Ranga belt (SRB) that yields concordia ages of 739 ± 3.4^20^, supporting the transitional stage (ca. 750–700 Ma). This is consistent with the nearby parts of the Shadli metavolcanics^19^ that are explained by the subduction–rift model, where arc magmatism (ca. 830–750 Ma) is followed by a proposed transitional stage (ca. 750–700 Ma), and subsequent plume-related rifting (< 700 Ma). These regional correlations, together with the observed geochemical transition, provide strong support for the proposed geodynamic evolution of the studied WRAM.

### Magmatic and post-magmatic mineralization

The Ranga metavolcanics show a complex history of both primary and secondary mineralization, reflecting the interplay of magmatic, hydrothermal, and supergene processes. This is evidenced through hydrothermally altered minerals and the textural and mineralogical diversity of primary and secondary sulfide mineralization in our metavolcanics. The Landsat-8 imagery and other remote sensing techniques (Figs. 4, 5; Supplementary Table 1 and Fig. 1) can delineate hydrothermal alteration in the studied WRAM. The effectiveness of BR transformations in Landsat-8 imagery for hydrothermal alteration mapping is well documented, supporting their use to distinguish primary magmatic and secondary alteration mineral assemblages. For instance, the 4/2, 6/5, and 6/7 band ratios are widely utilized to target key alteration indicators^44,134,135^. The 6/5 ratio enhances ferromagnesian minerals such as biotite and amphibole, typically associated with unaltered or weakly altered magmatic host rocks (Fig. 4d). In contrast, the 4/2 ratio highlights iron oxides that commonly form in supergene oxidation zones, reflecting post-magmatic overprinting (Fig. 4c). Meanwhile, the 6/7 ratio is sensitive to hydroxyl-bearing minerals (Fig. 4e), such as clays and micas, marking zones of argillic to phyllic alteration linked to hydrothermal fluid activity during the post-magmatic phase^44,134^. High PCA loadings on ASTER bands 2 and 4 may highlight iron oxides, which are typical indicators of post-magmatic supergene alteration. These iron-rich zones exhibit strong absorption in PC3 (-0.677) and appear as dark pixels (Fig. 5b), reflecting late-stage oxidation processes^53,54^. In contrast, Al(OH)-bearing minerals such as alunite, kaolinite, and illite, commonly formed during post-magmatic hydrothermal activity, show high reflectance in PC4 (0.574) and appear as white pixels (Fig. 5a), indicating zones of argillic to phyllic alteration^55,56^. Using the USGS spectral library alongside VNIR-SWIR reflectance data allows precise identification of key alteration minerals such as illite, kaolinite, and epidote (Fig. 5c), which are diagnostic of phyllic, argillic, and propylitic alteration zones, respectively. The alteration minerals and zones are commonly associated with post-magmatic hydrothermal systems^60–62^. Constrained energy minimization (CEM) spectral classification further enhances mineral mapping by isolating spectral signatures of economically significant minerals such as malachite, chalcopyrite, pyrite, hematite, magnetite, and sphalerite (Fig. 5c). Structural analysis through lineament extraction further substantiates the controls on mineralization. The predominance of NW–SE (Najd fault system) and NE–SW oriented lineaments, as evidenced by rose diagram distributions (Supplementary Fig. 1), suggests these structural trends serve as primary fluid pathways facilitating both magmatic-derived hydrothermal solution and post-magmatic alteration^64,65^. Their abundance and length underscore their geological significance, while the relative paucity of E–W and N–S lineaments points to an organized tectonic fabric influencing alteration and mineralization patterns.

The primary and secondary sulfide mineralization in the studied metavolcanics is petrographically and mineralogically investigated in this study (Fig. 3h). Primary sulfide mineralization is dominant in the mafic metavolcanics (Fig. 3h), particularly metabasalts, alkali metabasalts, metabasaltic andesites, and related lapilli metatuffs. Primary mineralization is exemplified by the presence of subhedral to euhedral pyrite grains (Fig. 7a), which typically form by magmatic or accumulate by early hydrothermal solution^136,137^. These primary sulfides can occur as disseminated euhedral crystals (Figs. 7c, d), such as chalcopyrite and sphalerite (Fig. 7f‒h), indicating deposition from magmatic-derived hydrothermally fluids at high temperatures and relatively reducing conditions^138,139^. Accumulation of primary sulfides in the post-magmatic stage is associated with widespread alteration features of silicate minerals. For example, if a sample has suffered from extensive alteration of plagioclase to sericite or saussurite and epidote (Fig. 6e‒f), it is possibly rich in sulfide mineralization. The elemental substitutions, such as Fe in sphalerite and Ni–Co–Ag in pyrite (Supplementary Table 3b), are attributed to magmatic-derived fluid interaction at high temperatures.

In addition, overprinting of primary sulfides by secondary processes is evidenced by the replacement of pyrite by goethite and the development of colloform goethite textures (Fig. 7b,e), especially pronounced in jasperoid samples, a product of supergene oxidation in the near-surface environment^140,141^. Such secondary mineralization reflects exposure of the ore body in the WRAM to meteoric waters, resulting in partial leaching, oxidation, and enrichment of sulfur and base metals in the supergene zone^142^. The Talc lenses hosted in metabasalts and metabasaltic andesites (Fig. 3d, e) typically develop through metamorphic and metasomatic processes involving the hydration and carbonation of magnesium-rich mafic minerals such as olivine, pyroxene, and amphibole^143^. Fault and shear zones with NW–SE (Najd system) and NE-SW trends (Supplementary Fig. 1) serve as major conduits for fluid circulation, promoting interaction between mafic minerals and water-rich fluids that lead to serpentinization, as evidenced by the coexistence of serpentine and talc (Fig. 6g-h). These structural patterns of the Najd fault and its conjugated trends (NE-SW and N-S) are prevalent in both the CED and SED and play a key role in controlling Fe-Ti ores, rare metals, sulfides, and gold mineralization in Egypt^17,144–146^. Subsequent infiltration of CO₂-rich fluids transforms serpentine into talc and talc–carbonate assemblages^147^. These alteration processes generally occur under low- to medium-grade metamorphic conditions, corresponding to the greenschist to lower amphibolite facies^148^. The presence of chrysocolla as green veinlets associated with talc in Atshan tremolite-talc rocks (Fig. 7i, Supplementary Fig. 2d-e) signifies supergene copper enrichment and the continued evolution of sulfide mineralization in the weathering environment^149^. In summary, the mineralogical and textural features (Fig. 7; Supplementary Fig. 2) of the studied WRAM demonstrate a paragenetic progression from high-T magmatic sulfides such as cubic pyrite–chalcopyrite–sphalerite mineralization to the widespread hydrothermal alteration and supergene oxidation, such as the occurrence of goethite, hematite, and jasperoids.

## Conclusions

1. Landsat-8 band ratios and ASTER BR in RGB successfully discriminate the studied metavolcanics, which are mainly metarhyolites, metadacites, and metabasalts (bimodal volcanism) with subordinate metaandesites and metabasaltic andesites, and their alteration zones. By using USGS spectral libraries along with the CEM technique, we successfully identify illite, kaolinite, and epidote, which represent phyllic, argillic, and propylitic alteration zones, respectively. Moreover, the ASTER spectral library is used to recognize malachite-bearing talc, sulfide mineralization (Fe–Cu–Zn–S), and iron-rich zones. The sulfide mineralization occurs as both disseminated and massive forms within metavolcanics and tremolite–talc rocks.
2. The lineament analysis shows that structural trends are dominantly oriented NW–SE and NE–SW with subordinate E–W and N–S directions. The prevailing NW–SE trend, together with its conjugate NE–SW and N–S lineaments, is consistent with the Najd fault system, which exerts a primary control on regional deformation and acts as a major shear corridors that enhance mineralizing fluid circulation during the subduction–rift transition.
3. Geochemical data of the studied metavolcanics indicate that the mafic rocks follow tholeiitic trends, whereas the felsic and intermediate to mafic varieties display calc-alkaline characteristics, reflecting bimodal magmatism. The alkali basalts display alkaline OIB-type signatures, implying localized intraplate mantle plume input. The calc-alkaline to tholeiitic metavolcanics are depleted in both REEs (ΣREEs < 20 ppm) and HFSEs (Nb, Ta, Ti) contents. In contrast, the alkali metabasalts are rich in REEs (ΣREEs:212.1 ppm on average), HFSEs, Ni (up to 245.1 ppm), and Cr (up to 337.8 ppm). Most metavolcanics were derived from a depleted mantle source similar to MORB and island-arc tholeiite, while alkali metabasalts originated from an enriched mantle source, likely related to mantle plume activity.
4. The calc-alkaline to tholeiitic volcanic types were generated by moderate degrees of partial melting (~ 10–20%) of a spinel lherzolite mantle source. In contrast, the alkali basalts most likely originated from low-degree partial melting (~ 5%) of a garnet lherzolite or garnet–spinel lherzolite source in deeper and more enriched mantle parts. These results reflect partial melting of a heterogeneous mantle source beneath the ANS.
5. Cpx chemistry of the alkali metabasalts indicates that their volcanic protolith crystallized at high temperatures of ~ 1000–1150 °C, and moderate pressures of ~ 2–5 kbar under high oxygen fugacity (*fO₂*) conditions.
6. The chemical data, magmatic affinity (tholeiitic, calc-alkaline, and alkaline magmas), and source of parent magmas, including the coexistence of OIB‑like and arc/MORB‑like basaltic melts, suggest the transition from the supra-subduction zone setting (e.g., the arc-related setting) of most Ranga-Atshan volcanics to the intraplate rifting setting of alkali basalts. Subduction to rifting transition, where the studied sample plot together with arc‑like signatures (MORB–arc) and within‑plate/OIB fields, marks a key phase of the geodynamic evolution of the Arabian–Nubian Shield.
7. The WRAM record a three-stage tectonomagmatic evolution from arc magmatism (tholeiitic–calc-alkaline) depleted in HFSEs through slab break-off with transitional compositions, to rifting-related magmatism dominated by alkaline, OIB-like basalts generated by mantle plume upwelling. This reflects progressive tectonic and mantle-source changes beneath the ANS.
8. Talc, malachite-bearing talc, and sulfide mineralization (e.g., VMS: Fe–Cu–Zn–S) follow a paragenetic sequence from magmatic sulfide assemblages to hydrothermal talc–carbonate alteration and supergene oxidation, forming malachite and chrysocolla. Pyrite, chalcopyrite, and sphalerite are the main sulfides, accompanied by chrysocolla as the only Si-bearing Cu mineral, reflecting hydrothermal mineralization with subsequent oxidation processes. Mineralization and hydrothermal alteration of the studied WRAM are structurally controlled by NW–SE and NE–SW shear zones, consistent with the Najd fault system.

## Supplementary Information

Supplementary Information 1.

Supplementary Information 2.

Supplementary Information 3.

Supplementary Information 4.

Supplementary Information 5.

## Data Availability

The datasets generated and/or analysed during the current study are available from the corresponding author on reasonable request.
