# Supplementary material for: Integrated remote sensing and geochemical data of Shadli mineralized metavolcanics (Egypt): mantle plume-driven magmatism during subduction–rift transition
Source: Sci Rep. 2026 Jul 14;16:22039. doi: 10.1038/s41598-026-60562-2 (PMC13369896; doi:10.1038/s41598-026-60562-2)
Supplement: Supplementary file 4 — Supplementary Information 4. [file 41598_2026_60562_MOESM4_ESM.docx]

**Integrated remote sensing and geochemical data of Shadli mineralized metavolcanics (Egypt): Mantle plume-driven magmatism during subduction–rift transition**

**Methodology**

For a detailed geological mapping of the Wadi Ranga area, Landsat-8 Operational Land Imager (OLI) and the Advanced Spaceborne Thermal Emission and Reflection Radiometer (ASTER) satellite images are used to discriminate rock units, structures, and alteration zones associated with copper and sulfide mineralization. The Landsat-8 OLI imagery encompasses a broad spectral range across nine spectral bands, featuring a spatial resolution of 30 meters for all bands, except for the 15-meter panchromatic band^1^, as shown in Supplementary Table 1a. The ASTER data covers an extensive spectrum with fourteen high-resolution bands that offer spatial, spectral, and radiometric capabilities. The visible and near-infrared (VNIR) bands have a spatial resolution of 15 meters, whereas the short-wave infrared (SWIR) bands are at 30 meters. The bands in the thermal infrared (TIR) regions have a spatial resolution of 90 meters. Each scene in the ASTER image covers 60 x 60 km^1^, as shown in Supplementary Table 1a. Nine ASTER VNIR–SWIR bands and seven spectral bands of Landsat-8 were acquired in February 2005 and July 2016^2,3^, respectively, and used for mapping in the current study area. The two datasets are subjected to both atmospheric and radiometric correction before the application of the various processing techniques of remote sensing using Envi version 5.3 software, Earth Resource Development Assessment System (ERDAS Imagine 2015), and ArcGIS 10.8. A FLAASH atmospheric correction technique has been applied to both ASTER and Landsat-8 to remove the atmospheric impacts and to change the radiance data to surface reflectance data. Also, Minimum Noise Reduction and inverse band ratio techniques have been applied on both ASTER and Landsat-8 data for noise removal and improvement of the data. Band Ratio (BR) and principal component (PC) (Supplementary Table 1b) have been used for the discrimination of rock units and alteration zones. Additionally, the Constrained Energy Minimization (CEM) and density slicing approaches were used for the best display of mineralized zones in the investigated area^4,5^. Finally, the automatic lineament extraction tool was employed to determine the common structural trend that controls the mineralized zones.

For petrography, sixty thin sections for rock samples, including forty-seven samples from Wadi Ranga and thirteen from Atshan metavolcanic rocks and related mineralization, were examined using reflected and transmitted light microscopes to determine their mineralogical and textural characteristics. Subsequently, thin sections were polished and coated with carbon at Chiba University in Japan for EPMA analyses and at Niigata University for EDS analyses. Additionally, a series of high-resolution photomicrographs was taken to highlight the key features of the Wadi Ranga metavolcanics and their mineralization at Kanazawa University.

Thirty rock samples from Wadi Ranga metavolcanics (twelve felsic, five intermediate-mafic, and thirteen mafic) were selected for major element analysis (Supplementary Table 2), based on petrographic studies, using X-ray Fluorescence (XRF) at the Geo Analytical Lab, Washington University, USA. Before analysis, the rock samples were crushed and ground into powder using an agate ball mill. The powdered samples were mixed with di-lithium tetraborate flux (in a 2:1 flux to rock ratio), fused at 1000 °C in a muffle furnace, and cooled. The resulting beads were further ground, re-fused, and polished on diamond laps to achieve a flat, smooth surface for analysis. Calibration was done using reference material 650CC from GSP2. Based on duplicate samples, the XRF analysis precision is generally better than 1% for most major elements. Loss on ignition (LOI) was measured by the weight difference after firing the samples at 1000 °C.

Additionally, some trace elements and REE (Supplementary Table 2) were obtained using Inductively Coupled Plasma Mass Spectrometry (ICP–MS) at the Geo Analytical Lab, Washington University, USA. Approximately 50 mg of each sample's powder was dissolved in acid-washed Teflon containers by refluxing in a 250 °C hot solution of nitric and hydrofluoric acid (3:1 ratio) for at least 8 hours. Calibration for instrument sensitivity was established using a blank fused bead from the same flux batch used for the unknown samples, along with USGS standards AGV–2 and RGM2. Additional USGS standards (DTS–2, BCR–1, G–2) were used for quality control. The analytical precision for trace elements is better than 5%, except for Ni, Cr, and Sc. The geochemical data were processed, and various diagrams were plotted using GCDkit v 3.6.0, Igpet 2010, and CorelDRAW 2017 software.

The major element analyses (Supplementary Table 3a) of silicate minerals, including plagioclase, amphibole, clinopyroxene, epidote, chlorite, and serpentine minerals from the WRAM and related talc rocks were performed by using an electron probe-micro analyzer (EPMA: JEOL JXA-8230) at Chiba University. The analytical condition was 15 kV acceleration voltage, 2.0 × 10-8 A beam current, and 3 μm beam diameter. Natural and synthetic standard minerals and metals were used for calibration. Additionally, the in-situ determination of trace and REE concentrations (Supplementary Table 3c) in plagioclase, amphibole and epidote from the WRAM was carried out using Laser-ablation inductively coupled plasma mass spectrometry (LA–ICP–MS) at Kanazawa University, Japan. The analysis utilized a 193 nm ArF excimer laser (MicroLas GeoLas Q-plus) with an Agilent 7500 S instrument. The procedures involved ablating 40-μm diameter spots at a rate of 6 Hz, with an energy density of 8 J/cm2 per pulse. Calibration was carried out based on internal and external standards; NIST 612 glass was used as an external standard, and ^29^Si was used as an internal standard based on the SiO_2_ concentration obtained by the electron microprobe. NIST 614 glass (secondary standard) was measured for quality control of each analysis. The accuracy and data quality based on the reference material (NIST 614) are high. The precision or reproducibility achieved was better than 5% for most elements, except for Sc, Cr, and Ni, for which it was better than 10%. Normalization of trace element data of the former minerals is carried out according to the standard chondrite (Cl) and primitive mantle (PM) of McDonough and Sun^6^.

Thin sections were polished and coated with carbon (60 thin polished) for Energy-Dispersive Spectroscopy (EDS) analyses. Comprehensive qualitative and semi-quantitative analyses of sixty points, encompassing Fe–Cu–Zn sulfide minerals, Fe–Ti oxides, copper-bearing talc and associated minerals (Supplementary Table 3b), were performed utilizing Scanning Electron Microscopy (SEM) equipped with Energy Dispersive Spectrometry (EDS) at Niigata University, Japan. The operating conditions were set to an accelerating voltage of 20 kV and a working distance of 10 mm. The EDS machines are mainly calibrated by using well‑known peaks from elements like aluminum and copper (e.g., Al Kα at about 1.49 keV and Cu Kα at about 8.04 keV). During the current sulfide mineral analyses by EDS in Niigata University (Japan), cobalt was used as a reference element during calibration to define characteristic X‑ray lines.

**References**

1. Abrams, M., Hook, S. & Ramachandran, B. J. J. p. l. ASTER user handbook, version 2. **4800**, 135 (2002).
2. U.S. Geological Survey. Landsat-8 OLI image, collection number 1. (2013).
3. NASA EOSDIS "LP DAAC". ASTER Level 1B Data Set Registered Radiance at the Sensor. doi:https://doi.org/10.5067/ASTER/AST_L1B.003 (2001).
4. Resmini, R., Kappus, M., Aldrich, W., Harsanyi, J. & Anderson, M. Mineral mapping with hyperspectral digital imagery collection experiment (HYDICE) sensor data at Cuprite, Nevada, USA. *International Journal of Remote Sensing* **18**, 1553–1570 (1997).
5. Farrand, W. H. & Harsanyi, J. C. Mapping the distribution of mine tailings in the Coeur d'Alene River Valley, Idaho, through the use of a constrained energy minimization technique. *Remote Sensing of Environment* **59**, 64–76 (1997).
6. McDonough, W. F. & Sun, S.-S. The composition of the Earth. *Chemical geology* **120**, 223–253 (1995).
