# Supplementary material for: Integrated remote sensing and geochemical data of Shadli mineralized metavolcanics (Egypt): mantle plume-driven magmatism during subduction–rift transition
Source: Sci Rep. 2026 Jul 14;16:22039. doi: 10.1038/s41598-026-60562-2 (PMC13369896; doi:10.1038/s41598-026-60562-2)
Supplement: Supplementary file 5 — Supplementary Information 5. [file 41598_2026_60562_MOESM5_ESM.pdf]

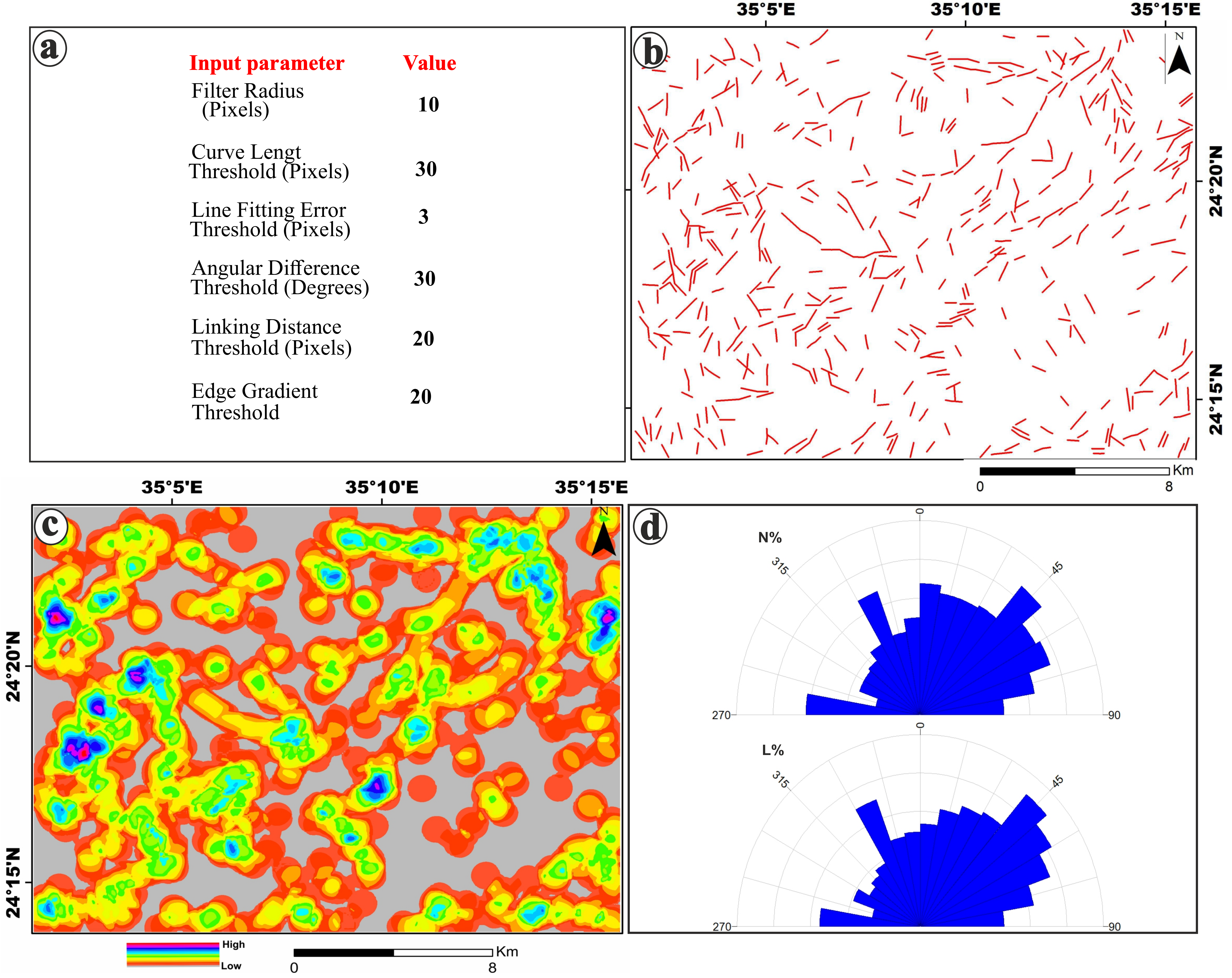


**Supplementary Fig. 1:** Lineaments extraction  (a) the used parameters in the automatic extraction process of lineament; (b) lineament map, the red lines represent lineament extracted using remote sensing techniques; (c) lineament density map of the study area; (d) Rose diagram showing the frequency distribution (N%) and the Length (L%) of the different structural trends in the study area.

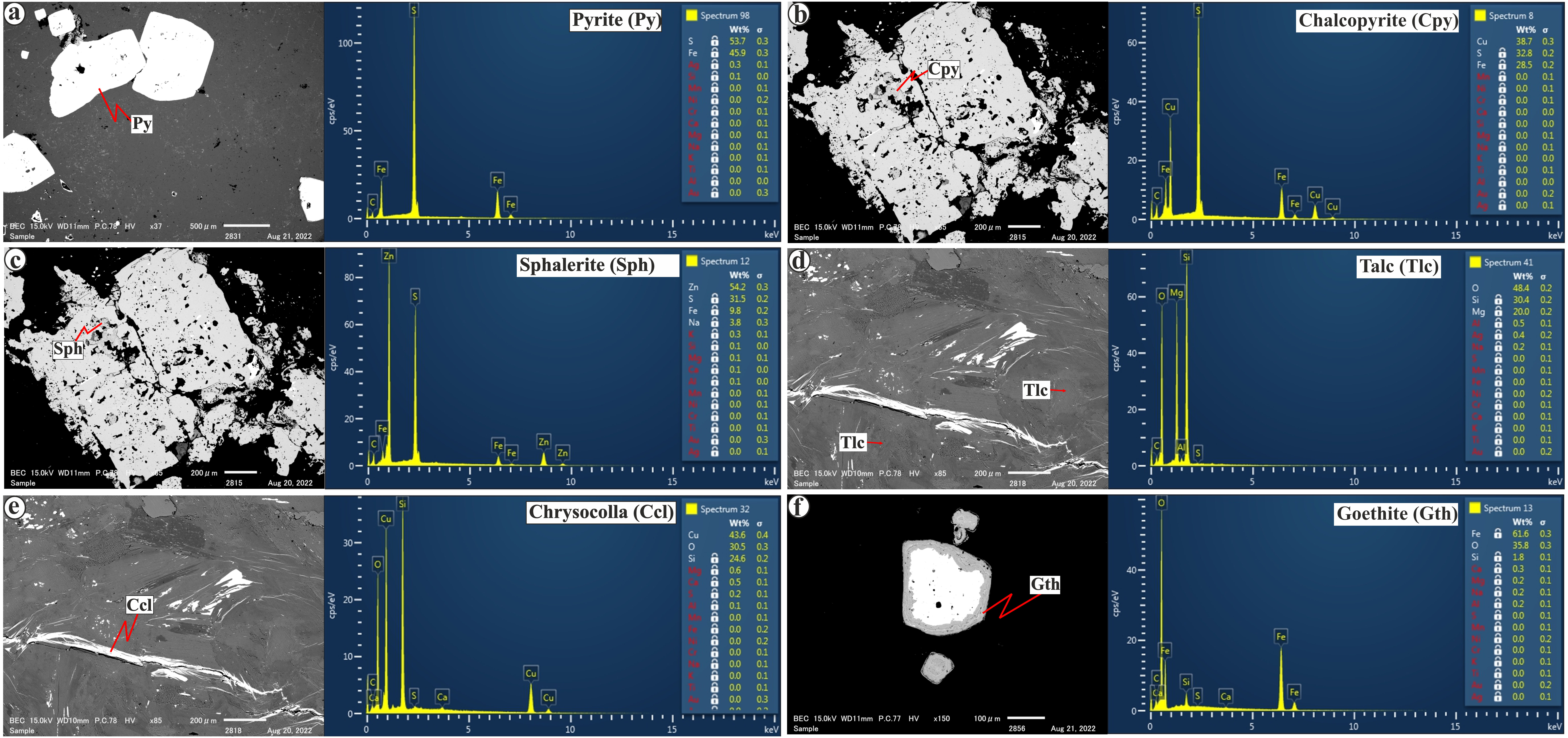


**Supplementary Fig. 2:** Scanning Electron Microscopy (SEM) images and EDS spectrum analysis of sulfides and associated minerals in WRAM MV. (a) pyrite; (b) chalcopyrite; (c) sphalerite; (d) talc; (e) chrysocolla; (f) goethite. Abbreviations: Pyrite (Py), Chalcopyrite (Cpy), sphalerite (Sph), goethite (Gth), Talc (Tlc), and Chrysocolla (Ccl).

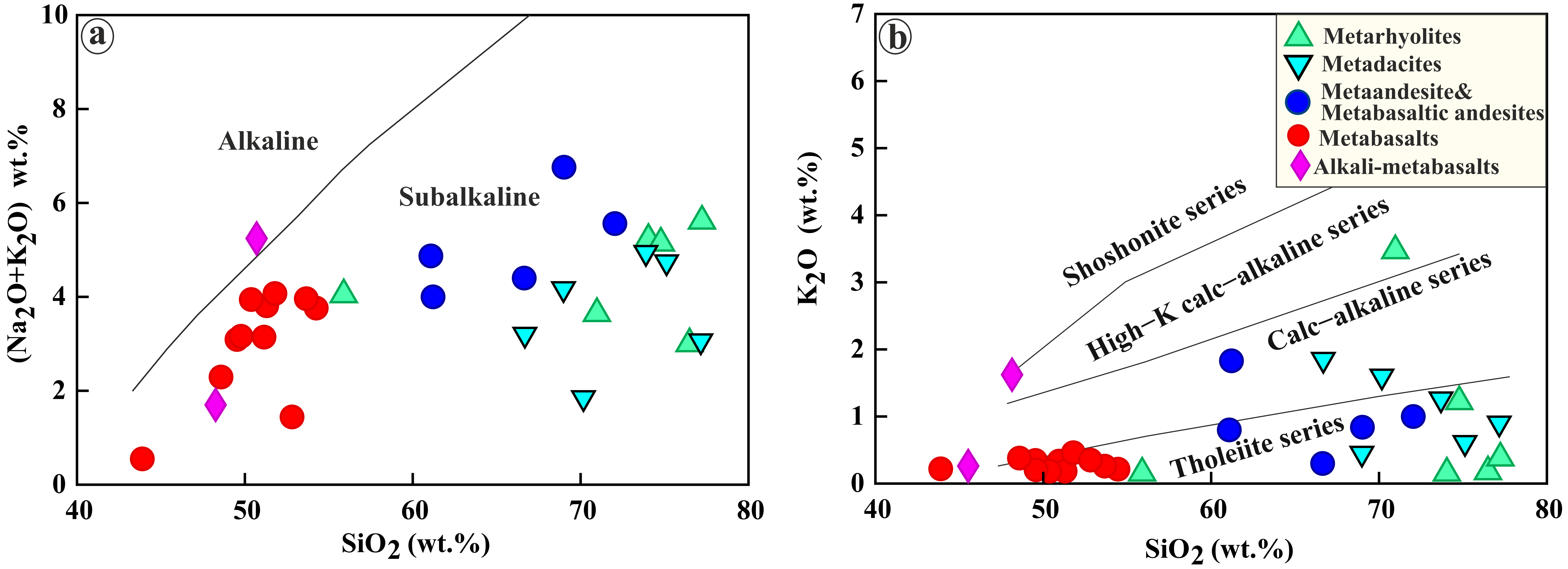


**Supplementary Fig. 3:** (a) ($Na_2O + K_2O$) vs. $SiO_2$ diagram[78] and (b) $K_2O$ vs. $SiO_2$ diagram[79]

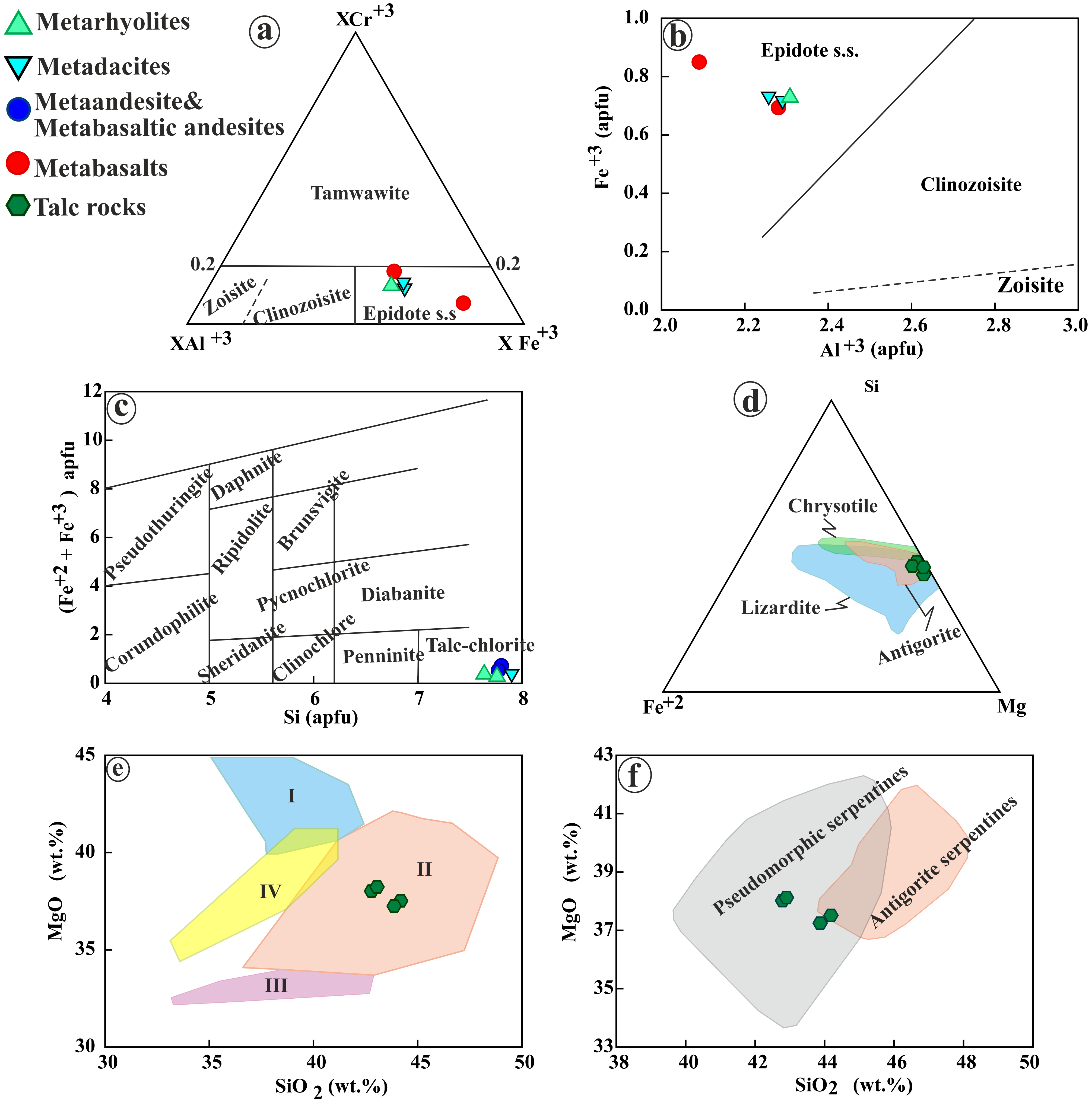


**Supplementary Fig. 4:** Mineral chemistry of clinopyroxene, epidote, chlorite, and serpentine minerals from WRAM MV and related rocks.(a, b) Epidote classification diagrams[90],(c) Si vs. Fe +Fe classification diagram of chlorite[91], (d–f) serpentine minerals classification diagrams from Atshan talc rocks[92,93].

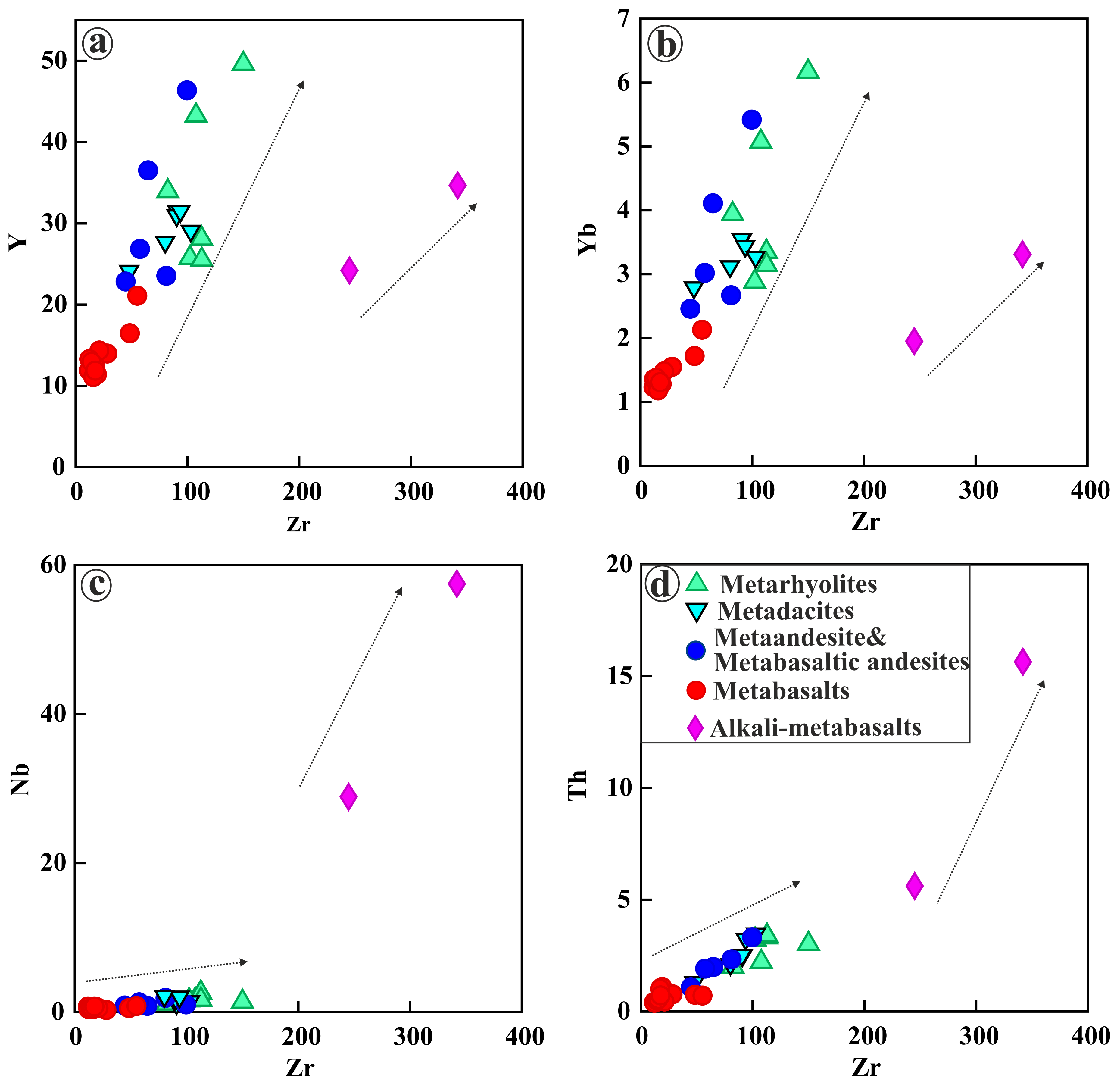


**Supplementary Fig. 5:** Variation diagrams of Zr versus some REEs and trace elements (Y, Yb, Nb, and Th).

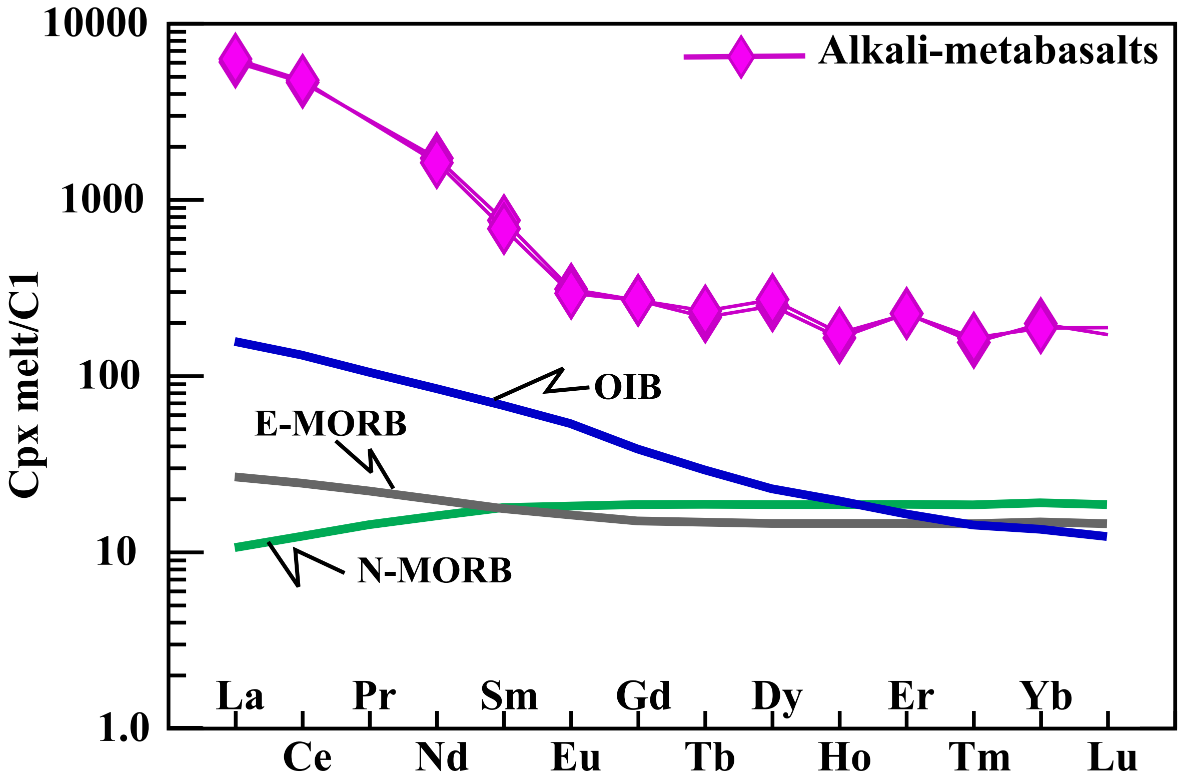


**Supplementary Fig. 6:** Chondrite-normalized REE patterns of melts inequilibrium with clinopyroxene. OIB, E-MORB, and N-MORB after McDonough and Sun[81]

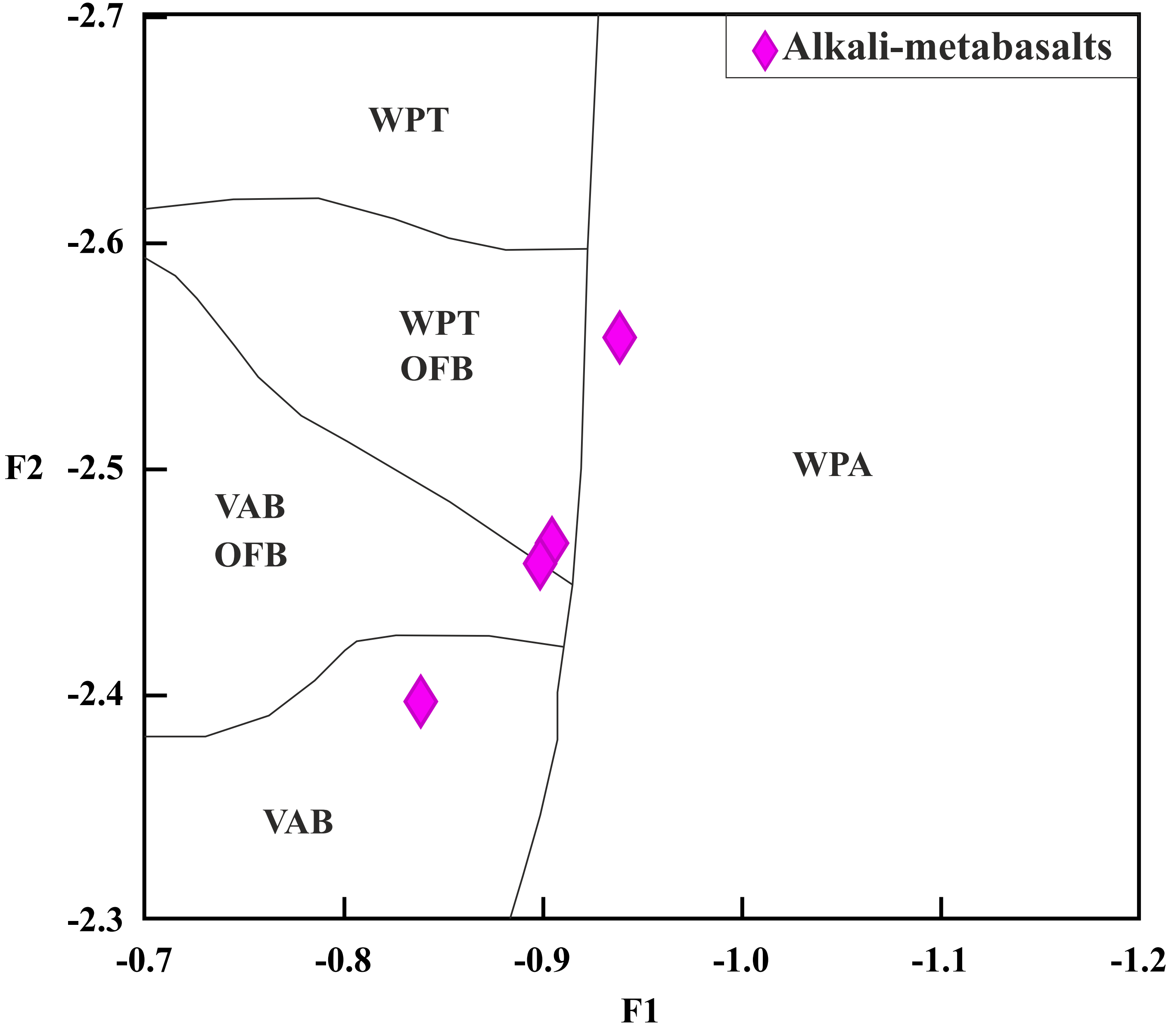


**Supplementary Fig. 7:** F1 vs. F2 diagram of Cpx from alkali-metabasalts[129].

## References


78. Irvine, T. N. & Baragar, W. A guide to the chemical classification of the common volcanic rocks. *Canadian journal of earth sciences* **8**, 523–548 (1971).

79. Peccerillo, A. & Taylor, S. Geochemistry of Eocene calc-alkaline volcanic rocks from the Kastamonu area, northern Turkey. *Contributions to mineralogy and petrology* **58**, 63–81 (1976).

81. Sun, S.-S. & McDonough, W. F. Chemical and isotopic systematics of oceanic basalts: implications for mantle composition and processes. *Geological Society, London, Special Publications* **42**, 313–345 (1989).

90. Franz, G. & Liebscher, A. Physical and chemical properties of the epidote minerals–An Introduction–. *Reviews in mineralogy and geochemistry* **56**, 1–81 (2004).

91. Hey, M. H. A new review of the chlorites. *Mineralogical Magazine and Journal of the Mineralogical Society* **30**, 277–292 (1954).

92. D'Antonio, M. & Kristensen, M. Serpentine and brucite of ultramafic clasts from the South Chamorro Seamount (Ocean Drilling Program Leg 195, Site 1200): inferences for the serpentinization of the Mariana forearc mantle. *Mineralogical Magazine* **68**, 887–904 (2004).

93. Wicks, F. J. & Plant, A. Electron microprobe and X-ray microbeam studies of serpentine textures. *Canadian Mineralogist* **17**, 785–830 (1979).

129. Nisbet, E. G. & Pearce, J. A. Clinopyroxene composition in mafic lavas from different tectonic settings. *Contributions to mineralogy and petrology* **63**, 149–160 (1977).
